# Resting Heart Rate and Associations With Clinical Measures From the Project Baseline Health Study: Observational Study

**DOI:** 10.2196/60493

**Published:** 2024-12-20

**Authors:** Kent Y Feng, Sarah A Short, Sohrab Saeb, Megan K Carroll, Christoph B Olivier, Edgar P Simard, Susan Swope, Donna Williams, Julie Eckstrand, Neha Pagidipati, Svati H Shah, Adrian F Hernandez, Kenneth W Mahaffey

**Affiliations:** 1 Stanford Center for Clinical Research Department of Medicine Stanford University School of Medicine Stanford, CA United States; 2 Verily Life Sciences South San Francisco, CA United States; 3 Cardiovascular Clinical Research Center, Department of Cardiology and Angiology, University Heart Center Freiburg Bad Krozingen, Faculty of Medicine University of Freiburg Freiburg Germany; 4 Duke University School of Medicine Durham, NC United States

**Keywords:** resting heart rate, wearable devices, remote monitoring, physiology, PBHS, Project Baseline Health Study, Verily Study Watch, heart rate, observational study, cohort study, wearables, electrocardiogram, regression analyses, socioeconomic status, medical condition, vital signs, laboratory assessments, physical function, electronic health, eHealth

## Abstract

**Background:**

Though widely used, resting heart rate (RHR), as measured by a wearable device, has not been previously evaluated in a large cohort against a variety of important baseline characteristics.

**Objective:**

This study aimed to assess the validity of the RHR measured by a wearable device compared against the gold standard of ECG (electrocardiography), and assess the relationships between device-measured RHR and a broad range of clinical characteristics.

**Methods:**

The Project Baseline Health Study (PHBS) captured detailed demographic, occupational, social, lifestyle, and clinical data to generate a deeply phenotyped cohort. We selected an analysis cohort within it, which included participants who had RHR determined by both ECG and the Verily Study Watch (VSW). We examined the correlation between these simultaneous RHR measures and assessed the relationship between VSW RHR and a range of baseline characteristics, including demographic, clinical, laboratory, and functional assessments.

**Results:**

From the overall PBHS cohort (N=2502), 875 (35%) participants entered the analysis cohort (mean age 50.9, SD 16.5 years; n=519, 59% female and n=356, 41% male). The mean and SD of VSW RHR was 66.6 (SD 11.2) beats per minute (bpm) for female participants and 64.4 (SD 12.3) bpm for male participants. There was excellent reliability between the two measures of RHR (ECG and VSW) with an intraclass correlation coefficient of 0.946. On univariate analyses, female and male participants had similar baseline characteristics that trended with higher VSW RHR: lack of health care insurance (both *P*<.05), higher BMI (both *P*<.001), higher C-reactive protein (both *P*<.001), presence of type 2 diabetes mellitus (both *P*<.001) and higher World Health Organization Disability Assessment Schedule (WHODAS) 2.0 score (both *P*<.001) were associated with higher RHR. On regression analyses, within each domain of baseline characteristics (demographics and socioeconomic status, medical conditions, vitals, physical function, laboratory assessments, and patient-reported outcomes), different characteristics were associated with VSW RHR in female and male participants.

**Conclusions:**

RHR determined by the VSW had an excellent correlation with that determined by ECG. Participants with higher VSW RHR had similar trends in socioeconomic status, medical conditions, vitals, laboratory assessments, physical function, and patient-reported outcomes irrespective of sex. However, within each domain of baseline characteristics, different characteristics were most associated with VSW RHR in female and male participants.

**Trial Registration:**

ClinicalTrials.gov NCT03154346; https://clinicaltrials.gov/study/NCT03154346

## Introduction

Resting heart rate (RHR) has been extensively studied in healthy individuals and those with specific disease states such as cardiovascular disease (CVD) [1,2]. Increasing RHR is linked to the development of CVD risk factors such as diabetes mellitus and hypertension and is implicated as an important prognostic factor in those with CVD and cancer [3,4]. Due to these links with important clinical outcomes such as the development of disease and mortality, RHR and RHR trends are of high interest to clinicians and patients alike and have become highly accessible, particularly with the recent ubiquity of wearable devices capable of recording heart rate (HR) and even detecting concerning arrhythmias such as atrial fibrillation [5].

Traditionally, RHR is determined through clinical measurements during physical examinations as well as electrocardiography (ECG), and ambulatory devices. In the recent decade, wearable devices have become increasingly popular; many have the capability to track fitness levels with a variety of metrics, including steps, HR, and sleep. Commercially available devices have been shown to be accurate in measuring HR and steps, and studies suggest that wearable devices may improve physical activity [6-8].

The Project Baseline Health Study (PBHS) was a prospective, multicenter, longitudinal cohort study launched in 2017 to establish a comprehensive reference health state using a wide range of modalities, evaluate different technologies in measuring disease trajectory and participant diversity, and share this information with both scientists and participants. The PBHS enrolled 2502 participants to include a broad range of healthy individuals with varying disease risks (specifically CVD, breast/ovarian cancer, and lung cancer), as well as those with known disease diagnoses. The PBHS provides an opportunity to describe and assess RHR using a wearable device (Verily Study Watch [VSW]) in a contemporary population and to do so in a comprehensive and more continuous manner than previously done [9]. Previous studies have limited comparisons with clinical measurements or have small sample sizes focused on specific disease states [10-12]. The design of the PBHS allows for an extensive analysis of RHR as they relate to multimodal clinical data collected from remote and in-person visits in a deeply phenotyped cohort, allowing a unique opportunity to explore potentially significant relationships. In this exploratory study, we aimed to (1) identify an analysis cohort within the PBHS and compare baseline characteristics with the overall study cohort at large, (2) validate the VSW’s determination of RHR (VSW RHR) by comparing against the gold standard of RHR by ECG, and (3) assess the relationships between VSW RHR and a broad range of baseline clinical characteristics.

## Methods

### Overview

The design of the PBHS has been previously described [9].

### Participants

PBHS participants were selected from an online registry in which participants entered basic demographic data so that the initial target cohort could be adequately established [9]. Ultimately, 2502 participants were included. The inclusion criteria for the registry were age ≥18 years, residency in the United States, ability to speak and read English, willingness to provide health information, and ability to interact with certain study activities using a personal smartphone/device. As one of the overarching goals of PBHS is to understand disease progression in the United States, the cohort was designed so that 60% of the enrolled population in each age strata had ~60% higher risk relative to the participants of the same age and sex for atherosclerotic cardiovascular disease, lung cancer, and/or breast or ovarian cancer.

### Measurements and Definitions

#### Study Assessments

PBHS participants underwent a deep phenotyping process, with extensive multimodal assessments during enrollment to measure their health characteristics, including demographics, vitals, laboratory, functional testing, imaging, surveys, and wearable sensor data from the VSW, an investigational medical device used in medical research and clinical care. For this study, baseline characteristics, as listed in Tables S1-S3 in Multimedia Appendix 1, were selected for each participant and were chosen in this exploratory work due to their ubiquity in clinical practice and physiological relevance to RHR.

#### Resting Heart Rate

Baseline RHR measurements were determined with 2 different techniques: in-clinic ECG RHR and VSW RHR. During the enrollment study site visit, a 12-lead ECG was recorded (Mortara ELI 250/250C), and HR from the computerized interpretation of the ECG was computed as the ECG RHR. An ECG was considered “Excellent” or “Good” when all 12 leads were analyzable, and either no noise/artifact or minimal noise/artifact (respectively) were noted; only ECG readings that met these criteria were considered.

VSW RHR was determined using a proprietary study wrist-wearable device, which was an integral part of the continuous assessments of PBHS. Participants were encouraged to wear it consistently during the entire study duration. The VSW captures biological signals through several sensors, including photoplethysmography (PPG) at 30 Hz and accelerometry at 30 Hz. It also provides several derived metrics using proprietary algorithms that process these signals. In this study, we use the following derived metrics:

- PPG Interbeat Intervals (IBI), which measure the time interval between PPG-derived heartbeats in milliseconds. The IBIs are calculated at each heartbeat, and each IBI value is also accompanied by a binary quality metric (“good” vs “bad” quality). To determine the quality of IBIs, in this study, we use the “jump distance” metric, which is defined as the following for each sample i: where *I_i_* is the IBI value in milliseconds at sample *i*. When the jump distance is smaller than 100 milliseconds, we label that IBI as having “good” quality and otherwise as having “bad” quality. The reason is that very high jump distance values indicate the presence of artifacts or the failure of the PPG peak detection algorithm. The threshold value of 100 milliseconds was chosen as the optimal value in a trade-off between heart rate error and coverage on an internally collected dataset.
- Actigraphy counts, which estimate the level of physical activity and are calculated every 30 seconds.
- On-wrist states, which indicate whether the VSW was worn or not, are computed every 1 minute and every time the on-wrist state changes.

**Figure 1:**
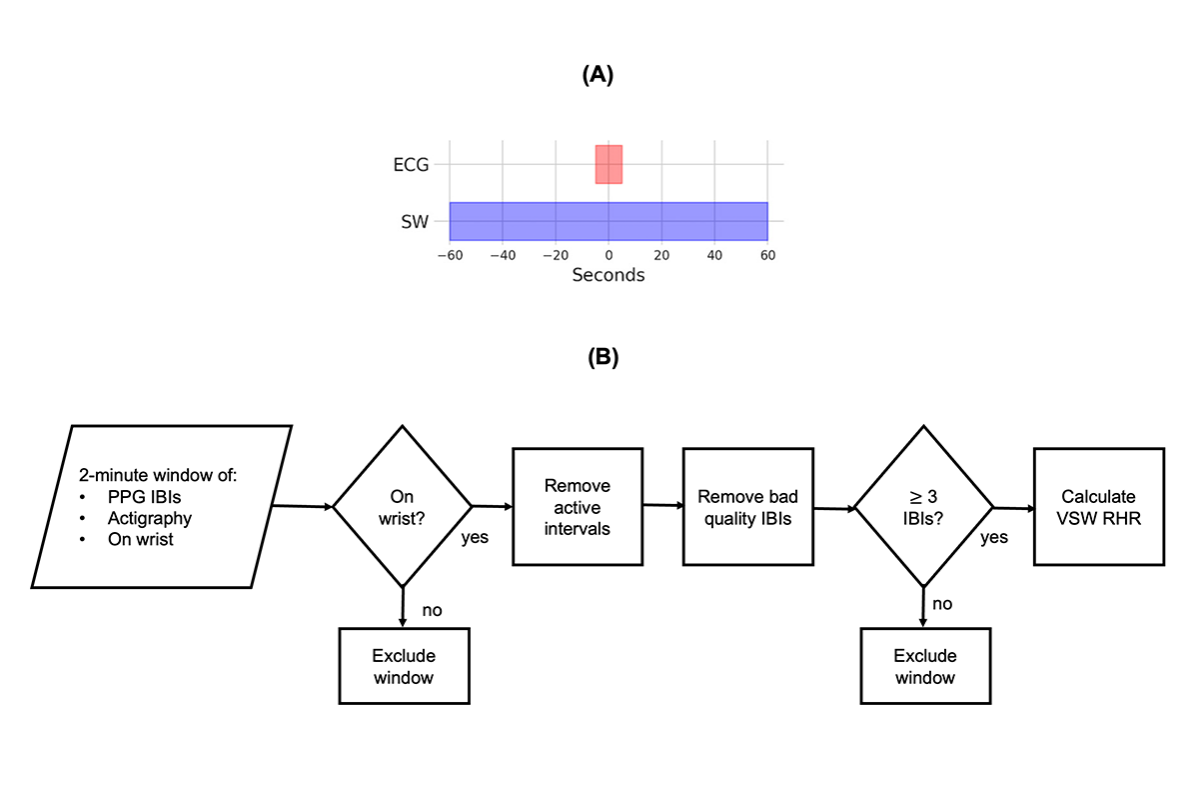
Verily Study Watch resting heart rate (VSW RHR) determination during the Project Baseline Health Study (PBHS) procedures. (A) Relative placement in time of the 2-minute VSW data acquisition window against the backdrop of the 10-second ECG acquisition window. (B) Flowchart showing the processing steps to calculate the VSW RHR for each participant. ECG: electrocardiogram; SW: study watch; PPG: photoplethysmography; IBI: interbeat interval; VSW: Verily Study Watch; RHR: resting heart rate.

Since the goal of this analysis was to compare the RHR estimated by VSW to ECG RHR, we used the VSW sensor data captured during the ECG RHR measurement in order to evaluate the performance of the VSW RHR. Thus, we gathered VSW data using a 2-minute measurement window centered at the middle of the ECG acquisition period, as shown in Figure 1A.

The processing steps to calculate the VSW RHR for each participant are shown in Figure 1B. First, we gathered PPG IBIs, actigraphy counts, and on-wrist states in the 2-minute window mentioned above. Then, we excluded 2-minute windows containing any off-wrist states, and we removed the IBIs associated with active intervals from the window (defined as any 30-second interval with a non-zero actigraphy count value, which we define as “Active”). Those intervals for which there was a zero actigraphy count were defined as “Still.” Finally, we removed the “bad” quality IBIs from the 2-minute window. If the remaining number of IBIs was less than 3, we excluded the participant; otherwise, we calculated the VSW RHR from the remaining IBIs as the following:



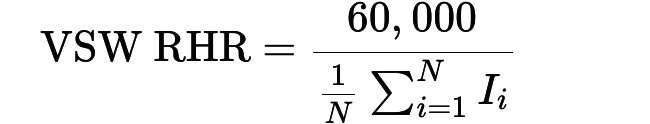



Where *I_i_* is the *i*th IBI value (milliseconds) in the 2-minute window, and N is the number of IBI values in the window.

#### Mean Daily Steps in the First 30 Days

Mean daily steps in the first 30 days of the study were calculated for each participant using previously validated step counts captured by the VSW RHR [13,14]. Specifically, daily step count values were averaged across the 30 days following enrollment, only considering the days during which the participant wore the VSW for at least 10 hours.

### Analysis Cohort

**Figure 2:**
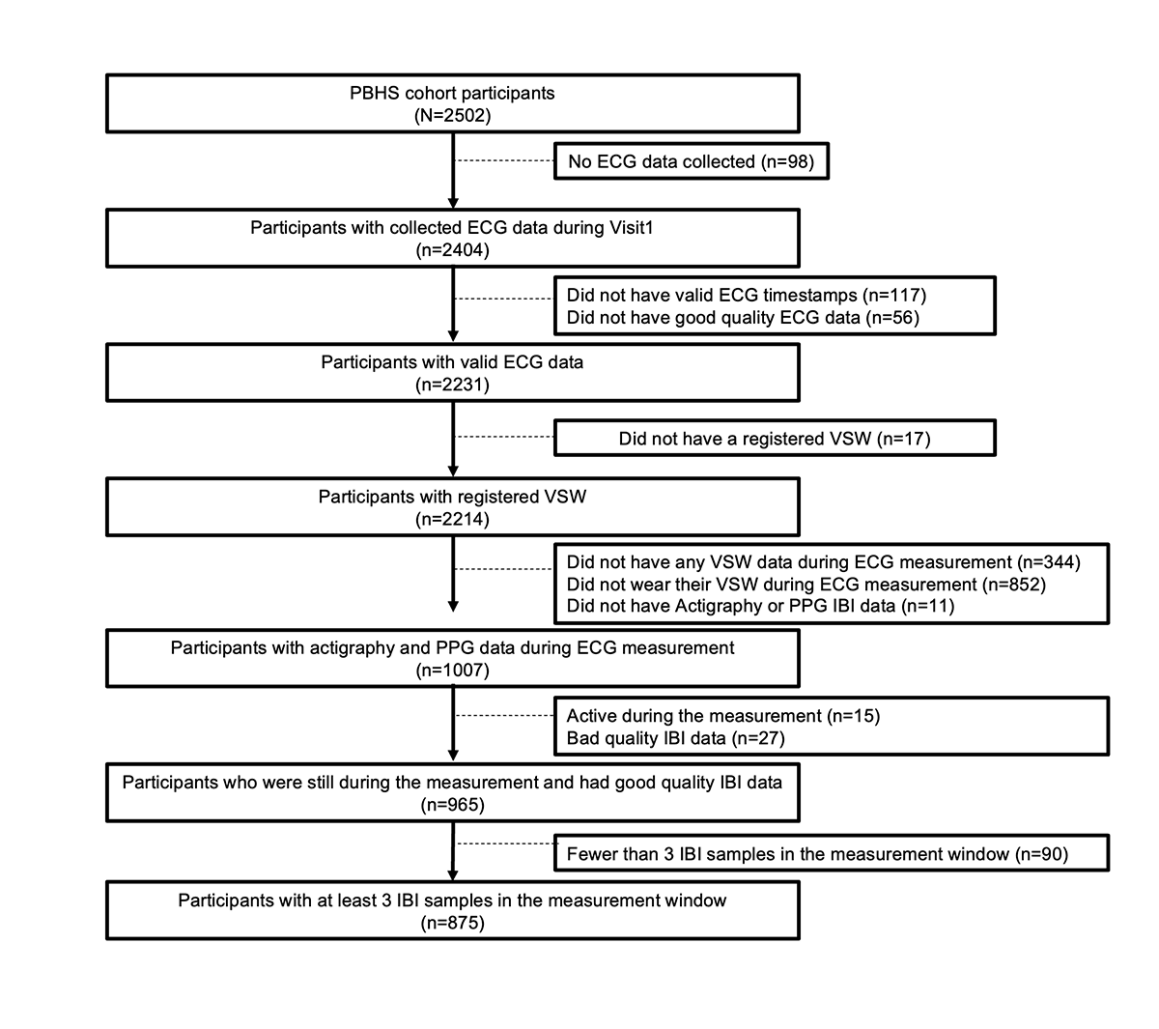
Analysis cohort flowchart. This flow chart details the creation of the eventual analysis cohort (n=875), originating from the full PBHS cohort. PBHS: Project Baseline Health Study; ECG: electrocardiogram; PPG: photoplethysmography; IBI: interbeat interval; VSW: Verily Study Watch.

For this analysis, the cohort included only participants who fulfilled the following criteria: (1) recorded ECG RHR during the initial onsite visit and (2) concurrent RHR as recorded by the VSW. Additional exclusion criteria were applied during the VSW RHR calculation procedure, as described in the previous section. Inclusion and exclusion criteria are further described in Figure 2.

### Study Watch Validity Analysis

To evaluate the validity of VSW RHR measurements, we first compared them to ECG RHR using the intraclass correlation (ICC) coefficient across the participants [15]. In addition, we calculated the bias in VSW RHR compared with ECG RHR, which is defined as the mean of the difference between the two measurements (VSW RHR and ECG RHR) across all participants. To determine how bias changed as a function of ECG RHR, we fitted a linear model to predict the measurement difference using ECG RHR as input, and we measured the slope of the fitted model. For both bias and slope values, we calculated the 95% CIs using bootstrapping (1000 bootstraps). We repeated these analyses for each of the male and female subgroups.

### Statistical Analysis

Descriptive statistics were calculated for selected demographics and other baseline characteristics. Categorical variables were reported as the number of participants with corresponding percentages, and continuous variables were reported as mean and SD.

For use in statistical testing and regression modeling, categorical variables were translated into a series of 1/0 “dummy” variables, representing each level of each predictor variable versus all other levels as the reference.

Tests for trend were used to evaluate the relationship between each characteristic and ordinal category of VSW RHR, separately for males and females. Analyses were stratified by sex due to well-established baseline differences in RHR by sex and to document sex-related differences in overall baseline characteristics. Baseline characteristic differences across RHR percentiles were not statistically compared between males and females. In addition, 3 VSW RHR categories were created using sex-specific percentile cut points: 0-25th, 25th-75th, and 75th-100th. The Cochran-Armitage Trend Test was used to evaluate binary variables (including the “dummy” indicator variables created for each level of categorical variables), and Spearman rank correlation was used to evaluate continuous variables.

Associations with VSW RHR among candidate baseline characteristics were identified using multivariable linear regression models. Before modeling, missing data were imputed using 5 rounds of multiple imputation using chained equations methods with predictive mean matching. Box-Cox transformations were used to approximate a normal distribution for continuous variables (laboratory values, vitals, and physical function measures). In addition to observed age, age-squared was added to the list of baseline variables to account for the inverted U-shaped relationship between age and VSW RHR.

All baseline characteristics (more details in Tables S1-S3 in Multimedia Appendix 1) were included as candidate variables in the least absolute shrinkage and selection operator (LASSO) regression models; no characteristics captured beyond the baseline period were included. Baseline characteristics were grouped into domains: (1) demographics and socioeconomic status (SES), (2) medical conditions, (3) vitals and physical function, (4) laboratory assessments, and (5) patient-reported outcomes (PROs); separate models were built for each domain and separately for male and female. Elastic net (ENET) regularization methods were used to fit regression models. In order to address the multiply-imputed data, a stacked objective function (sENET) method was used, with 5-fold cross-validation to penalize and select regression coefficients [16,17]. Due to limitations in computational power, ENET alpha values were restricted to 0.5 or 1, where α=1 equates to a LASSO regression. All predictors were standardized before use in modeling as required for LASSO and ENET methods.

### Ethical Considerations

The study was approved by a central institutional review board (IRB; Western IRB: approval tracking number 20170163, work order number 1-1506365-1) and the IRB at each of the participating institutions (Stanford University, Duke University, and the California Health and Longevity Institute). The PBHS was registered in ClinicalTrials.gov (identifier NCT03154346).

Informed consent was obtained from all participants enrolled in PBHS. Participants received small compensation for study visit–related time and expenses. This report is based on analyses of deidentified data.

## Results

### Analysis Cohort Compared With the Overall PBHS Cohort

Using the criteria as described in Figure 2, the analysis cohort consisted of 875 participants: 519 (59%) female and 356 (41%) male. Selected baseline characteristics of the analysis cohort and the PBHS cohort are shown in Table 1.

**Table 1:** Selected baseline characteristics of the PBHS cohort and the analysis cohort. Overall, the cohorts are similar in baseline characteristics.

				PBHS^a^ cohort (N=2502)		Analysis cohort (n=875)
**Demographics**						
	Mean age at enrollment, years (SD)			50 (17.2)		50.9 (16.5)
	Female, n (%)			1375 (55)		519 (59.3)
	**Race, n (%)**					
		White	1582 (63.2)		575 (65.7)	
		Black	400 (16)		138 (15.8)	
		Asian	260 (10.4)		80 (9.1)	
		Other	259 (10.4)		82 (9.4)	
	Hispanic, n (%)			290 (11.6)		98 (11.2)
**Socioeconomic status, n (%)**						
	Married			1116 (44.6)		433 (49.5)
	Employed			1523 (60.9)		528 (60.3)
	Current or former smoker			881 (35.2)		331 (37.8)
**Medical conditions, n (%)**						
	Asthma			371 (14.8)		124 (14.2)
	Diabetes, type 2			276 (11.0)		112 (12.8)
	Generalized anxiety disorder			327 (13.1)		121 (13.8)
	GERD^b^			424 (16.9)		176 (20.1)
	Hypertension			675 (27)		262 (29.9)
	Hypercholesterolemia			314 (12.5)		118 (13.5)
	Major depressive disorder			354 (14.1)		142 (16.2)
	Migraines			306 (12.2)		116 (13.3)
	Osteoarthritis			477 (19.1)		179 (20.5)
	Sleep apnea			245 (9.8)		88 (10.1)
**Vitals**						
	Mean systolic BP^c^ (SD)			123.4 (16)		125 (15.5)
	Mean diastolic BP (SD)			75.9 (9.9)		77.4 (9.9)
	Mean BMI (SD)			28.4 (6.9)		29.4 (7.1)
**Physical performance**						
	Mean 6-minute walk distance, meters (SD)			474.5 (82.7)		475.4 (88.2)
	Mean left ventricular ejection fraction (SD)			58.7 (4.2)		58.6 (4.5)
**Laboratory findings**						
	Hemoglobin A_1c_, mean (SD)			5.7 (1)		5.8 (1.1)
	Hemoglobin (g/dL), mean (SD)			14.2 (1.3)		14.1 (1.3)
	White blood cell count (thousand/mcL), mean (SD)			6.4 (1.9)		6.6 (1.9)
	MDRD^d^ (eGFR^e^), mean (SD)			88.3 (20.4)		87.5 (21.1)
	C-reactive protein (mg/L), mean (SD)			2.9 (5.9)		3.4 (7.2)
**Patient-reported outcomes**						
	PHQ-9^f^ score, mean (SD)			3.7 (4.2)		3.9 (4.3)
	GAD-7^g^ score, mean (SD)			3.2 (4.1)		3.3 (4.2)

^a^PBHS: Project Baseline Health Study.

^b^GERD: gastro-esophageal reflux disease.

^c^BP: blood pressure.

^d^MDRD: modification of diet in renal disease.

^e^eGFR: estimated glomerular filtration rate.

^f^PHQ-9: Patient Health Questionnaire-9.

^g^GAD-7: Generalized Anxiety Disorder-7 Scale.

### Study Watch Validity

**Figure 3:**
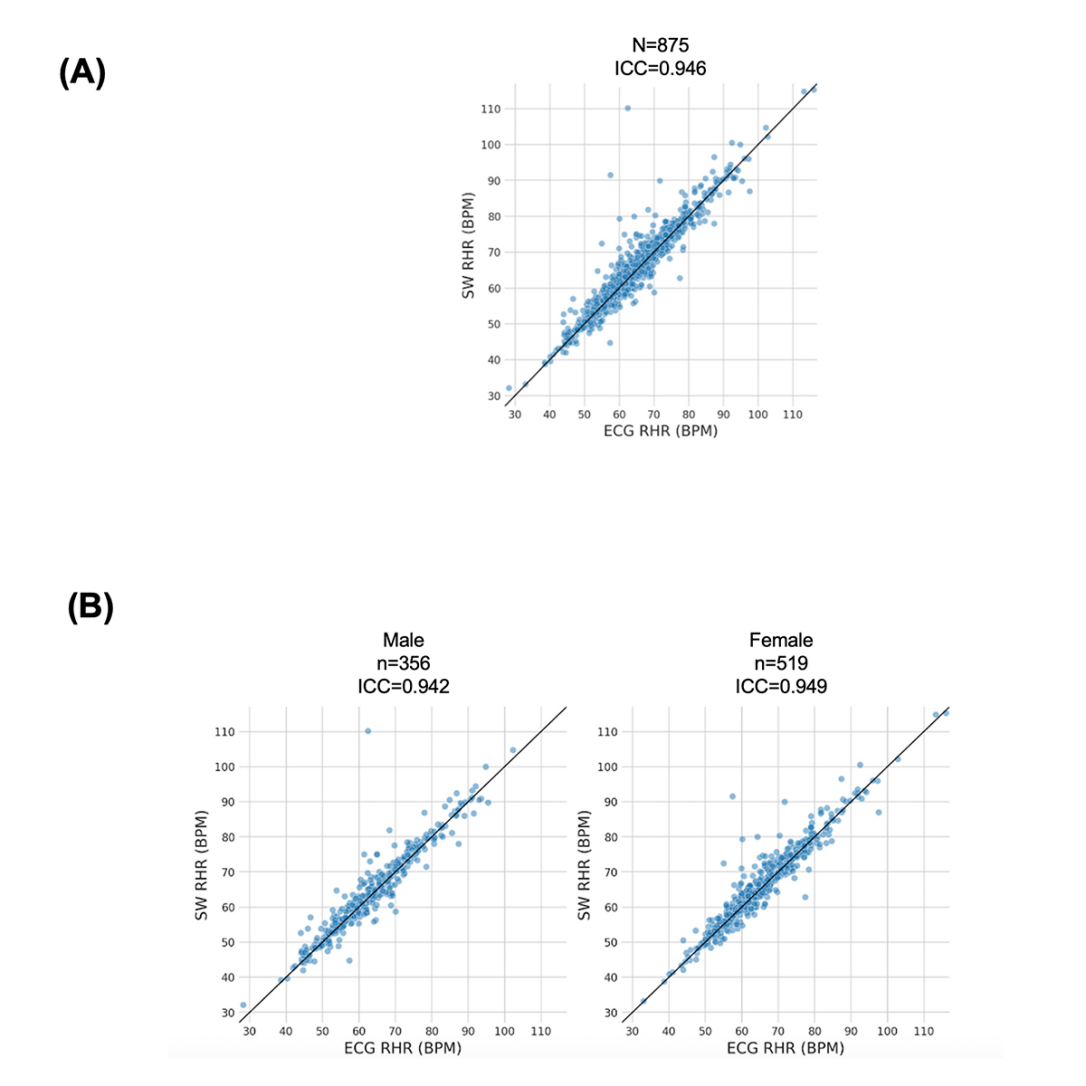
Correlation between baseline ECG-based and Study Watch measured RHR in this analysis cohort (within the PBHS) for (A) all participants and (B) male (left) and female (right) participants separately. Each dot corresponds to one participant. There is excellent overall reliability between ECG RHR and VSW RHR (ICC=0.946) and within each of the male (0.942) and female (0.949) subgroups. BPM: beats per minute; ECG: electrocardiogram; ICC: intraclass correlation coefficient; RHR: resting heart rate; SW: study watch.

The comparison of the RHR by ECG with VSW is shown in Figure 3A. There was excellent reliability between the 2 measures, with an intraclass correlation coefficient of 0.946 (Figure 3A). This reliability remained excellent within each of the male and female subgroups (Figure 3B). An agreement plot between RHR by ECG and VSW of all participants also showed high consistency between the two measures (Figure S1 in Multimedia Appendix 1).

We also estimated the bias in the VSW measurement of RHR compared to the ECG RHR. The overall bias was 0.76 BPM (95% CI 0.52-1.00), which indicated a small but significant positive bias, meaning that VSW was slightly overestimating the RHR when compared with ECG-based RHR. We had a similar result in male and female subgroups, with a bias of 0.70 (95% CI 0.29-1.14) and 0.80 (95% CI 0.51-1.13), respectively.

Finally, we evaluated how bias changed as a function of ECG RHR using the slope of a fitted linear model (Figure S2A in Multimedia Appendix 1). The resulting slope was –0.029 (95% CI –0.047 to –0.010), which indicated a small but significant negative slope, meaning that for the ECG RHR values on the lower end, VSW overestimated the RHR, while on the higher end, it underestimated the RHR. We had a similar result for each of the male and female subgroups, with a slope of –0.030 (95% CI –0.057 to –0.003) and –0.028 (95% CI –0.056 to –0.003), respectively (Figure S2B in Multimedia Appendix 1).

### Analysis Cohort Baseline Characteristics by Sex and Resting Heart Rate (Age-Adjusted)

**Figure 4:**
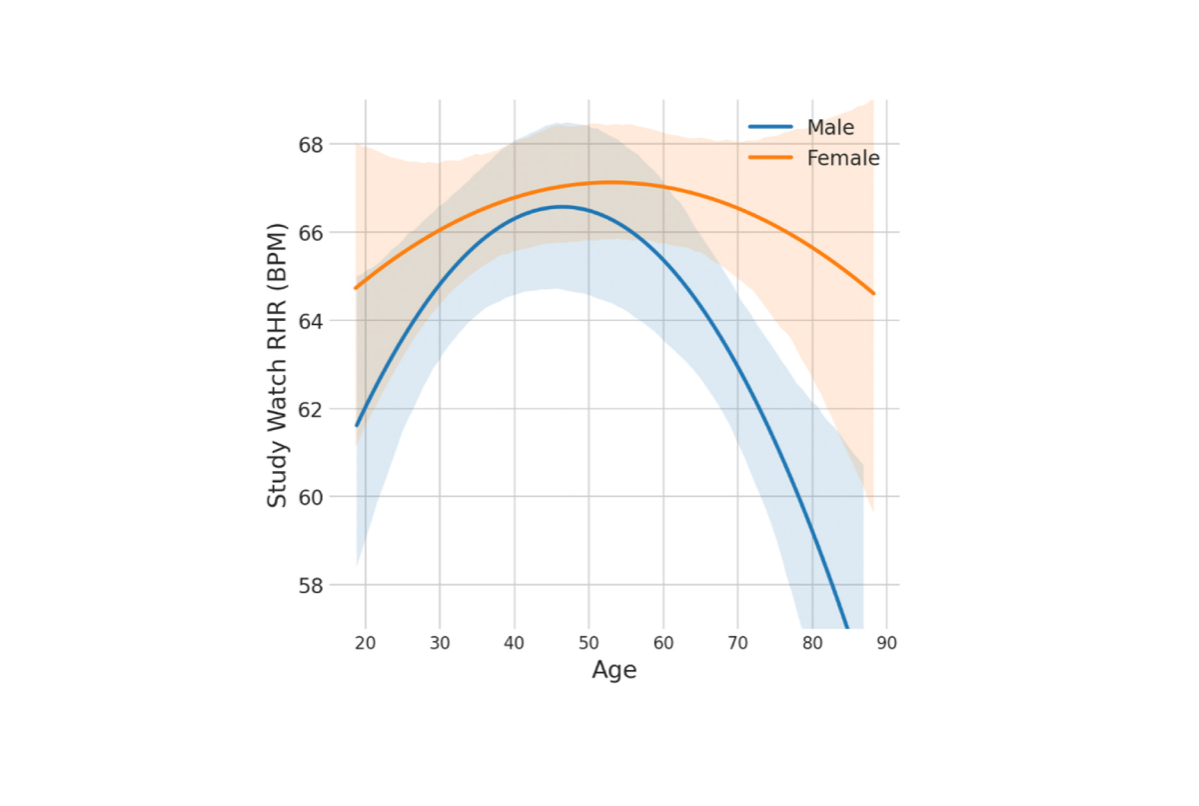
Baseline study watch resting heart rate by age and sex in this analysis cohort (within the PBHS). A U-shaped curve was observed for both female and male participants when Verily Study Watch resting heart rate (VSW RHR) was plotted against age. The lines show fitted quadratic models for female and male data separately. The shaded areas show the 95% CIs of the models. BPM: beats per minute; RHR: resting heart rate.

The VSW RHR as a function of age and sex is shown in Figure 4, with both curves demonstrating the expected upside-down U-shaped relationship [10]. The mean and SD of VSW RHR was 66.6 (SD 11.2) beats per minute (bpm) for female participants and 64.4 (SD 12.3) bpm for male participants. For ECG RHR, the mean and SD were 65.8 (SD 10.9) bpm for females and 63.7 (SD 12.0) bpm for males.

The study cohort was then separated by sex and stratified by VSW RHR to assess for trends of selected baseline characteristics: demographics and SES (Tables 2 and 3; vitals, physical function, and laboratory assessments (Tables 4 and 5); and medical conditions and participant-reported outcomes (PROs; Tables 6 and 7). Full variable comparison lists are included in Tables S1-S3 in Multimedia Appendix 1.

**Table 2:** Analysis cohort, female participants (within the PBHS): Selected demographics and socioeconomic status at baseline, stratified by Verily Study Watch resting heart rate (VSW RHR) percentile.

			VSW^a^ RHR^b^ percentile^c^						
			0-25th (n=130)		25-75th (n=259)		75-100th (n=130)		*P* value
Mean age, years (SD)			48.7 (16.3)		51.1 (16.1)		49.3 (15.8)		.76
**Race, n (%)**									
	White	93 (71.5)		164 (63.3)		83 (63.8)		.19	
	Black	18 (13.8)		44 (17)		26 (20)		.19	
	Asian	10 (7.7)		27 (10.4)		6 (4.6)		.37	
	Other (NHPI^d^, AIAN^e^, Other)	9 (6.9)		24 (9.3)		15 (11.5)		.20	
Hispanic ethnicity, n (%)			14 (10.8)		40 (15.4)		17 (13.1)		.59
**Education level, n (%)**									
	High school or less	11 (9.7)		21 (9.5)		24 (20.9)		.01	
	Any college	65 (57.5)		143 (64.7)		66 (57.4)		.98	
	Graduate degree or higher	37 (32.7)		57 (25.8)		25 (21.7)		.06	
**Income (US $), n (%)**									
	<100,000	58 (51.3)		123 (55.7)		82 (71.3)		.002	
	>100,000	46 (40.7)		82 (37.1)		25 (21.7)		.003	
**Marital status, n (%)**									
	Married	63 (55.8)		131 (59.3)		50 (43.5)		.06	
	Divorced or separated	21 (18.6)		36 (16.3)		22 (19.1)		.91	
	Single	24 (21.2)		43 (19.5)		33 (28.7)		.17	
	Widowed	3 (2.7)		7 (3.2)		10 (8.7)		.03	
**Employment status, n (%)**									
	Employed or homemaker	92 (74.2)		164 (68.3)		69 (57.5)		.01	
	Unemployed	6 (4.8)		17 (7.1)		24 (20)		<.001	
	Retired	19 (15.3)		52 (21.7)		23 (19.2)		.44	
	Student	5 (4)		4 (1.7)		2 (1.7)		.21	
Insured (health insurance, yes), n (%)			109 (96.5)		202 (91.4)		101 (89.4)		.048
**Smoking status, n (%)**									
	Current smoker	12 (9.2)		41 (15.8)		31 (23.8)		.001	
	Former smoker	27 (20.8)		54 (20.8)		24 (18.5)		.64	
	Never smoker	91 (70)		164 (63.3)		75 (57.7)		.04	

^a^VSW: Verily study watch.

^b^RHR: resting heart rate.

^c^Percentile cut points for females: 25th=59.38 bpm; 75th=73.66 bpm. Shading for significant observations. To generate *P* values for tests for trend, the Cochran-Armitage was used to evaluate binary variables, including ‘dummy’ indicator variables created for each level of categorical variables, and Spearman Rank Correlation was used to evaluate continuous variables.

^d^NHPI: Native Hawaiian, and Pacific Islander.

^e^AIAN: American Indians and Alaska Natives.

**Table 3:** Analysis cohort, male participants (within the PBHS): Selected demographics and socioeconomic status at baseline, stratified by Verily Study Watch resting heart rate (VSW RHR) percentile.

		VSW^a^ RHR^b^ percentile^c^			
		0-25th (n=89)	25-75th (n=178)	75-100th (n=89)	*P* value
Mean age, years (SD)		55.5 (16.6)	50.7 (18.5)	51.6 (14.3)	.11
**Race, n (%)**					
	White	71 (79.8)	107 (60.1)	57 (64.0)	.03
	Black	11 (12.4)	24 (13.5)	15 (16.9)	.39
	Asian	4 (4.5)	24 (13.5)	9 (10.1)	.22
	Other (NHPI^d^, AIAN^e^, Other)	3 (3.4)	23 (12.9)	8 (9.0)	.20
Hispanic ethnicity, n (%)		6 (6.7)	12 (6.7)	9 (10.1)	.40
**Education level, n (%)**					
	High school or less	7 (9.7)	16 (10.7)	12 (15.4)	.28
	Any college	34 (47.2)	74 (49.7)	48 (61.5)	.08
	Graduate degree or higher	31 (43.1)	59 (39.6)	18 (23.1)	.01
**Income (US $), n (%)**					
	<100,000	34 (47.2)	80 (53.7)	41 (52.6)	.53
	>100,000	35 (48.6)	64 (43.0)	28 (35.9)	.12
**Marital status, n (%)**					
	Married	57 (79.2)	86 (57.7)	46 (59.0)	.01
	Divorced or separated	5 (6.9)	11 (7.4)	7 (9.0)	.64
	Single	9 (12.5)	49 (32.9)	22 (28.2)	.04
	Widowed	0 (0.0)	1 (0.7)	2 (2.6)	.28
**Employment status, n (%)**					
	Employed or homemaker	43 (53.8)	103 (65.6)	57 (67.1)	.08
	Unemployed	9 (11.2)	9 (5.7)	11 (12.9)	.67
	Retired	28 (35.0)	40 (25.5)	16 (18.8)	.02
	Student	0 (0.0)	4 (2.5)	1 (1.2)	.80
Insured (health insurance, yes), n (%)		69 (95.8)	137 (91.9)	67 (85.9)	.03
**Smoking status, n (%)**					
	Current smoker	16 (18.0)	22 (12.4)	17 (19.1)	.84
	Former smoker	21 (23.6)	41 (23.0)	25 (28.1)	.49
	Never smoker	52 (58.4)	115 (64.6)	47 (52.8)	.44

^a^VSW: Verily study watch.

^b^RHR: resting heart rate.

^c^Percentile cutpoints for males: 25th=55.50 bpm; 75th=72.25 bpm. Shading for significant observations. To generate *P* values for tests for trend, the Cochran-Armitage was used to evaluate binary variables, including ‘dummy’ indicator variables created for each level of categorical variables, and Spearman Rank Correlation was used to evaluate continuous variables.

^d^NHPI: Native Hawaiian, and Pacific Islander.

^e^AIAN: American Indians and Alaska Natives.

**Table 4:** Analysis cohort, female participants (within the PBHS): Selected vitals, physical function, and labs at baseline, stratified by Verily Study Watch resting heart rate (VSW RHR) percentile.

		VSW^a^ RHR^b^ percentile^c^			
		0-25th (n=130)	25-75th (n=259)	75-100th (n=130)	*P* value
**Vitals**					
	Systolic blood pressure, mean (SD)	119.5 (15.2)	122.6 (15.5)	126.5 (15.6)	<.001
	Diastolic blood pressure, mean (SD)	73.2 (8.3)	76.0 (9.2)	81.1 (10.0)	<.001
	Waist circumference (cm), mean (SD)	85.3 (14.4)	89.8 (15.9)	98.5 (18.0)	<.001
	BMI, mean (SD)	27.1 (6.4)	28.5 (6.7)	32.9 (8.4)	<.001
**Physical function**					
	6-minute walk distance (m), mean (SD)	498.1 (82.7)	469.3 (81.9)	433.8 (93.0)	<.001
	10-meter walk speed (seconds), mean (SD)	2.0 (0.6)	1.9 (0.4)	1.8 (0.5)	.001
	Handgrip, mean (SD)	28.9 (6.9)	28.1 (6.9)	27.4 (7.0)	.23
	Leg balance time (seconds), mean (SD)	44.3 (20.6)	39.8 (22.1)	37.8 (23.1)	.02
	Sit-rise score, mean (SD)	7.5 (2.3)	6.9 (2.5)	7.0 (2.4)	.11
	30-second chair stand, mean (SD)	14.8 (4.7)	13.9 (5.0)	12.9 (4.3)	.002
	Ejection fraction at rest (%), mean (SD)	59.0 (3.6)	59.4 (4.3)	58.5 (5.4)	.33
	Coronary calcium score, mean (SD)	66.6 (214.3)	60.9 (250.0)	76.6 (249.1)	.03
	Ankle brachial index abnormal, n (%)	4 (3.1)	10 (3.9)	3 (2.5)	.79
	FEV_1_/FVC^d^, mean (SD)	0.8 (0.1)	0.8 (0.1)	0.8 (0.1)	.25
	Daily steps in the first 30 days, mean (SD)	8360 (2990)	8040 (3187)	6865 (3243)	.0001
**Laboratory values**					
	Hemoglobin (g/dL), mean (SD)	13.5 (1.0)	13.5 (1.2)	13.7 (1.2)	.10
	Serum creatinine (mg/dL), mean (SD)	0.8 (0.1)	0.8 (0.2)	0.8 (0.2)	.19
	HDL^e^ (mg/dL), mean (SD)	66.4 (18.4)	64.3 (20.5)	57.2 (14.4)	<.001
	LDL^f^ (mg/dL), mean (SD)	96.0 (29.1)	105.5 (33.9)	105.8 (30.4)	.02
	HbA_1c_ (%), mean (SD)	5.4 (0.7)	5.6 (0.8)	6.0 (1.5)	<.001
	C-reactive protein (mg/L), mean (SD)	2.3 (4.1)	3.3 (5.1)	5.6 (8.2)	<.001
	Blood glucose (mg/dL), mean (SD)	88.7 (19.0)	94.1 (27.2)	108.9 (54.6)	<.001
	Hematocrit (%), mean (SD)	41.2 (2.9)	41.2 (3.4)	42.0 (3.5)	.047
	Platelet count (per µL), mean (SD)	249,836 (56,779)	259,269 (61,124)	278,790 (61,889)	<.001
	WBC^g^ count (thousand/µL), mean (SD)	6.2 (1.6)	6.5 (1.8)	7.5 (2.2)	<.001
	Sodium (mEq/L), mean (SD)	139.0 (1.8)	138.8 (2.1)	138.6 (2.2)	.15
	GFR MDRD^h^ (mL/min), mean (SD)	86.2 (19.2)	87.5 (20.1)	91.0 (24.9)	.19
	TSH^i^ (mIU/L), mean (SD)	1.5 (0.8)	1.6 (1.2)	1.6 (0.9)	.35

^a^VSW: Verily study watch.

^b^RHR: resting heart rate.

^c^Percentile cutpoints for females: 25th=59.38 bpm; 75th=73.66 bpm. Shading for significant observations. To generate *P* values for tests for trend, the Cochran-Armitage was used to evaluate binary variables, including ‘dummy’ indicator variables created for each level of categorical variables, and Spearman Rank Correlation was used to evaluate continuous variables.

^d^FEV1/FVC=forced expiratory volume in 1 s /forced vital capacity.

^e^HDL: high-density lipoprotein.

^f^LDL: low-density lipoprotein.

^g^WBC: white blood cell.

^h^GFR MDRD: glomerular filtration rate, modification of diet in renal disease.

^i^TSH: thyroid-stimulating hormone.

Similar trends were seen in female and male participants. For instance, from an SES standpoint, those with higher baseline VSW RHR were more likely to have lower household income, less likely to be married, less likely to have health care insurance, and more likely to be smokers.

Medical conditions such as major depressive disorder, type 2 diabetes mellitus, hypertension, and sleep apnea were also more common in those with higher VSW RHR.

Participants with higher VSW RHR tended to have higher systolic and diastolic blood pressures, BMI, and waist circumference.

In terms of laboratory assessments, those with higher VSW RHR tended to have hemoglobin A_1c_ %, C-reactive protein levels, and white blood cell counts.

Participants with higher VSW RHR had shorter 6-minute walk distances and fewer mean daily steps, as recorded by the VSW.

From a PRO standpoint, participants with higher VSW RHR had higher Patient Health Questionnaire-9 (PHQ-9) scores and World Health Organization Disability Assessment Schedule (WHODAS 2.0) scores.

**Table 5:** Analysis cohort, male participants (within the PBHS): Selected vitals, physical function, and labs at baseline, stratified by Verily Study Watch resting heart rate (VSW RHR) percentile.

		VSW^a^ RHR^b^ percentile^c^			
		0-25th (n=89)	25-75th (n=178)	75-100th (n=89)	*P* value
**Vitals**					
	Systolic blood pressure, mean (SD)	127.7 (16.2)	128.1 (14.1)	129.3 (14.3)	.37
	Diastolic blood pressure, mean (SD)	76.1 (10.4)	78.3 (9.7)	81.2 (10.1)	.002
	Waist circumference (cm), mean (SD)	95.5 (12.3)	98.7 (15.7)	108.4 (18.5)	<.001
	BMI, mean (SD)	27.6 (4.5)	29.3 (5.7)	32.5 (8.5)	<.001
**Physical function**					
	6-minute walk distance (m), (SD)	501.5 (83.1)	490.2 (89.8)	465.2 (84.6)	.002
	10-meter walk speed, mean (SD)	2.1 (0.6)	2.1 (0.6)	1.9 (0.5)	.021
	Handgrip, mean (SD)	46.0 (9.4)	44.5 (10.6)	42.4 (10.3)	.088
	Leg balance time (seconds), mean (SD)	38.4 (22.8)	37.7 (23.1)	30.6 (22.6)	.01
	Sit-rise score, mean (SD)	7.5 (2.1)	7.0 (2.3)	6.7 (2.3)	.02
	30-second chair stand, mean (SD)	15.4 (5.3)	14.9 (5.5)	13.4 (4.4)	.002
	Ejection fraction at rest (%), mean (SD)	58.2 (3.7)	57.7 (4.7)	58.7 (4.4)	.76
	Coronary calcium score, mean (SD)	361.8 (1012.1)	254.6 (653.9)	209.5 (632.9)	.10
	Ankle brachial index abnormal, n (%)	3 (3.5%)	7 (3.9%)	2 (2.3%)	.65
	FEV_1_/FVC^d^, mean (SD)	0.7 (0.1)	0.8 (0.1)	0.8 (0.1)	.003
	Daily steps in the first 30 days, mean (SD)	8970 (3994)	8565 (3537)	7869 (4120)	.07
**Laboratory findings**					
	Hemoglobin (g/dL), mean (SD)	14.8 (0.9)	14.9 (1.0)	15.1 (1.1)	.02
	Serum creatinine (mg/dL), mean (SD)	1.0 (0.2)	1.0 (0.3)	1.1 (0.5)	.95
	HDL^e^ (mg/dL), mean (SD)	54.3 (17.4)	48.3 (15.4)	43.9 (12.8)	<.001
	LDL^f^ (mg/dL), mean (SD)	93.3 (36.3)	95.0 (33.1)	101.6 (38.5)	.22
	HbA_1c_ (%), mean (SD)	5.5 (0.5)	5.7 (1.0)	6.5 (1.9)	.003
	C-reactive protein (mg/L), mean (SD)	3.0 (14.4)	2.5 (4.5)	4.6 (7.3)	<.001
	Blood glucose (mg/dL), mean (SD)	92.2 (12.4)	102.0 (35.9)	130.1 (72.8)	<.001
	Hematocrit (%), mean (SD)	44.7 (2.9)	45.2 (3.1)	45.8 (3.2)	.01
	Platelets (per µL), mean (SD)	212,529 (48,991)	228,567 (53,411)	248,264 (68,746)	<.001
	WBC^g^ count (thousand/µL), mean (SD)	6.2 (1.9)	6.2 (1.5)	6.9 (1.9)	.001
	Sodium (mEq/L), mean (SD)	139.4 (1.7)	138.9 (2.1)	138.6 (2.4)	.02
	GFR MDRD^h^ (mL/min), mean (SD)	84.3 (16.5)	88.2 (20.7)	86.6 (25.2)	.38
	TSH^i^ (mIU/L), mean (SD)	1.8 (1.0)	1.9 (1.1)	1.7 (0.9)	.71

^a^VSW: Verily study watch.

^b^RHR: resting heart rate.

^c^Percentile cutpoints for males: 25th=55.50 bpm; 75th=72.25 bpm. Shading for significant observations. To generate *P* values for tests for trend, the Cochran-Armitage was used to evaluate binary variables, including ‘dummy’ indicator variables created for each level of categorical variables, and Spearman Rank Correlation was used to evaluate continuous variables.

^e^HDL: high-density lipoprotein.

^f^LDL: low-density lipoprotein.

^g^WBC: white blood cell.

^h^GFR MDRD: glomerular filtration rate, modification of diet in renal disease.

^i^TSH: thyroid-stimulating hormone.

**Table 6:** Analysis cohort, female participants (within the PBHS): Selected medical conditions and participant-reported outcomes (PROs) at baseline, stratified by Verily Study Watch resting heart rate (VSW RHR) percentile.

		VSW^a^ RHR^b^ percentile^c^			
		0-25th (n=130)	25-75th (n=259)	75-100th (n=130)	*P* value
**Medical history, n (%)**					
	Asthma	16 (12.3)	35 (13.5)	23 (17.7)	.21
	Cataracts	12 (9.2)	38 (14.7)	22 (16.9)	.07
	Colon polyps	7 (5.4)	26 (10)	10 (7.7)	.50
	Major depressive disorder	15 (11.5)	43 (16.6)	29 (22.3)	.02
	Diabetes type 2	6 (4.6)	27 (10.4)	26 (20)	<.001
	GERD^d^	20 (15.4)	42 (16.2)	36 (27.7)	.01
	Hypertension	27 (20.8)	70 (27)	40 (30.8)	.07
	Hypercholesterolemia	14 (10.8)	40 (15.4)	13 (10)	.85
	Osteoarthritis	25 (19.2)	49 (18.9)	32 (24.6)	.28
	Sleep apnea	6 (4.6)	16 (6.2)	11 (8.5)	.20
**PRO scores** ^e^ **, mean (SD)**					
	Sheehan Disability Scale	2.9 (4.5)	2.7 (4.8)	5.0 (7.6)	.10
	PHQ-9^f^	3.4 (3.6)	3.6 (4)	5.4 (4.8)	<.001
	GAD-7^g^	3.2 (3.9)	3.4 (4.1)	4.1 (4.9)	.28
	WHODAS^h^ 2.0	2.2 (3.3)	3.0 (4.4)	5.0 (6.7)	<.001
	BRFSS ACE^i^	2.2 (2.2)	2.4 (2.3)	2.7 (2.6)	.24
	PROMIS^j^ pain intensity	6.0 (2.3)	6.1 (2.3)	7.0 (2.8)	.02
	PROMIS pain interference	10.2 (5.1)	10.5 (5.2)	12.2 (6.3)	.03
	PANAS^k^ positive affect	34.7 (6.4)	34.8 (7.0)	33.0 (7.3)	.08
	PANAS negative affect	15.6 (6.7)	15.5 (6.5)	15.0 (6.1)	.45
	Subjective happiness	21.6 (4.8)	21.7 (4.8)	20.9 (4.3)	.13
	Satisfaction with life	26.1 (6.6)	25.9 (6.3)	24.2 (7.4)	.04
	Perceived social support	70.1 (11.8)	66.8 (15.2)	66.9 (13.9)	.08
	AUDIT-C^l^	2.0 (1.5)	2.0 (1.9)	1.9 (1.8)	.35

^a^VSW: Verily study watch.

^b^RHR: resting heart rate.

^c^Percentile cutpoints for females: 25th=59.38 bpm; 75th=73.66 bpm. Shading for significant observations. To generate *P*-values for tests for trend, the Cochran-Armitage was used to evaluate binary variables, including ‘dummy’ indicator variables created for each level of categorical variables, and Spearman Rank Correlation was used to evaluate continuous variables.

^d^GERD: gastroesophageal reflux disease.

^e^PROs: patient-reported outcomes.

^f^PHQ-9: Patient Health Questionnaire-9.

^g^GAD-7: Generalized Anxiety Disorder-7 Scale.

^h^WHODAS: World Health Organization Disability Assessment Schedule.

^i^BRFSS ACE: Behavioral Risk Factor Surveillance System Adverse Childhood Experience.

^j^PROMIS: Patient-Reported Outcomes Measurement Information System.

^k^PANAS: Positive and Negative Affect Schedule.

^l^AUDIT-C: Alcohol Use Disorders Identification Test-Concise.

**Table 7:** Analysis cohort, male participants (within the PBHS): Selected medical conditions and participant-reported outcomes (PROs) at baseline, stratified by Verily Study Watch resting heart rate (VSW RHR) percentile.

		VSW^a^ RHR^b^ percentile^c^			
		0-25th (n=89)	25-75th (n=178)	75-100th (n=89)	*P* value
**Medical history, n (%)**					
	Asthma	12 (13.5)	22 (12.4)	16 (18)	.39
	Cataracts	14 (15.7)	28 (15.7)	5 (5.6)	.046
	Colon polyps	14 (15.7)	23 (12.9)	6 (6.7)	.07
	Major depressive disorder	8 (9)	28 (15.7)	19 (21.3)	.02
	Diabetes type 2	3 (3.4)	23 (12.9)	27 (30.3)	<.001
	GERD^d^	21 (23.6)	37 (20.8)	20 (22.5)	.86
	Hypertension	29 (32.6)	61 (34.3)	35 (39.3)	.35
	Hypercholesterolemia	15 (16.9)	27 (15.2)	9 (10.1)	.20
	Osteoarthritis	21 (23.6)	33 (18.5)	19 (21.3)	.71
	Sleep apnea	9 (10.1)	29 (16.3)	17 (19.1)	.10
**PRO scores** ^e^ **, mean (SD)**					
	Sheehan Disability Scale	3.3 (6.6)	3.1 (5.6)	4.2 (5.7)	.06
	PHQ-9^f^	3.2 (4.1)	3.8 (4.4)	4.6 (4.4)	.01
	GAD-7^g^	2.1 (3.2)	2.9 (4)	3.9 (4.8)	.02
	WHODAS 2.0^h^	2.2 (4.5)	3.2 (5.2)	4.4 (5.4)	<.001
	BRFSS ACE^i^	1.6 (1.9)	1.9 (2.2)	2.7 (2.6)	.01
	PROMIS^j^ pain intensity	6.1 (2.7)	6.4 (2.3)	6.5 (2.5)	.18
	PROMIS pain interference	10.0 (5.7)	10.6 (4.7)	12.4 (6.1)	.003
	PANAS^k^ positive affect score	35.5 (7.7)	33.7 (7.6)	32.1 (8.1)	.01
	PANAS negative affect	14.9 (5.7)	15.2 (5.9)	15.1 (5.8)	.93
	Subjective happiness	22.0 (4.3)	20.7 (4.8)	18.9 (4.6)	<.001
	Satisfaction with life	26.1 (6.6)	24.7 (6.7)	22.5 (6.2)	<.001
	Perceived social support	65.8 (13.8)	61.6 (16.7)	59.3 (14.5)	.003
	AUDIT-C^l^	2.3 (1.7)	2.1 (1.8)	1.8 (1.8)	.02

^a^VSW: Verily study watch.

^b^RHR: resting heart rate.

^c^Percentile cutpoints for males: 25th=55.50 bpm; 75th=72.25 bpm. Shading for significant observations. To generate *P* values for tests for trend, the Cochran-Armitage was used to evaluate binary variables, including ‘dummy’ indicator variables created for each level of categorical variables, and Spearman Rank Correlation was used to evaluate continuous variables.

^d^GERD: gastroesophageal reflux disease.

^e^PROs: patient-reported outcomes.

^f^PHQ-9: Patient Health Questionnaire-9.

^g^GAD-7: Generalized Anxiety Disorder-7 Scale.

^h^WHODAS: World Health Organization Disability Assessment Schedule.

^i^BRFSS ACE: Behavioral Risk Factor Surveillance System Adverse Childhood Experience.

^j^PROMIS: Patient-Reported Outcomes Measurement Information System.

^k^PANAS: Positive and Negative Affect Schedule.

^l^AUDIT-C: Alcohol Use Disorders Identification Test-Concise.

### Associations With VSW RHRs by Domain

**Figure 5:**
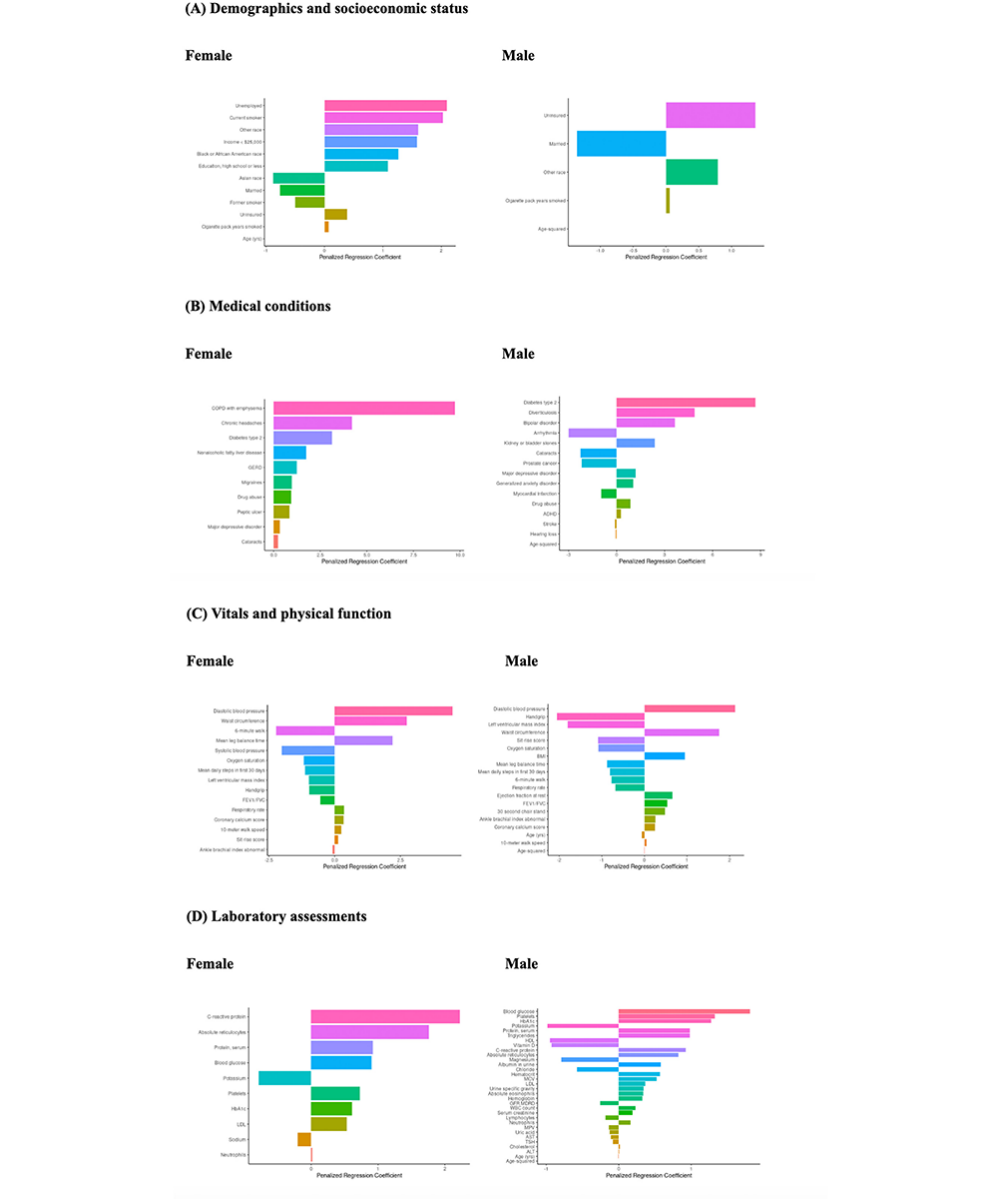
LASSO (least absolute shrinkage and selection operator) regressions. Regression analyses performed in this cohort (within the PBHS) suggest that, within each domain, different baseline characteristics are most associated with resting heart rate. All continuous measurements, ie, laboratory, vital, and physical function variables, were transformed by the Box-Cox method before analysis. All variables were standardized as required for penalized regression methodology. Within each domain of baseline characteristics, there were some characteristics that were more associated with RHR in female participants and others that were more associated with male participants. A higher-resolution version of this image is available in Multimedia Appendix 2.

The results of the sex-stratified sENET regression models are presented in Figure 5. Penalized regression coefficients reflect the relative strength and direction of each association based on standardized predictors. Within each domain of baseline characteristics (demographics and SES, medical conditions, vitals, physical function, laboratory assessments, and PROs), analyses showed that different characteristics were associated with VSW RHR in female and male participants. For instance, in the demographics and SES domain, unemployment had the highest association with VSW RHR in females, whereas lack of health insurance had the highest association in male participants. This was the case in the medical conditions, laboratory assessments, and PRO domains as well. For the vitals and physical function domain, diastolic blood pressure was the most associated characteristic with VSW RHR for both sexes.

## Discussion

### Principal Findings

These analyses from a large, deeply phenotyped population show (1) strong agreement between ECG-determined RHR and a proprietary VSW-determined RHR, (2) significant trends of VSW RHR with clinically important baseline characteristics, and (3) clinical baseline characteristics highly associated with VSW RHR. These findings demonstrate that, in a relatively heterogeneous cohort of participants, RHR can be measured easily and accurately using a wearable device and may be used in light of strong associations with clinically relevant baseline characteristics.

In the last decade, the use of consumer wearables with the ability to detect HR and arrhythmias such as atrial fibrillation has become increasingly available [5]. Despite their high accuracy in measuring HR at rest and the ubiquity of these devices in modern life, an in-clinic ECG is still the gold standard method to determine RHR [18]. In clinical research, a variety of methodologies are used depending on the availability of data and clinical feasibility [10,11,19,20]. In this study, we investigated the viability of the VSW in determining RHR by comparing it with RHR determined by ECG. Using PPG data combined with actigraphy data, we isolated periods of time when the participant wearing the VSW was not in motion during the time of ECG recording, thus allowing us to estimate RHR values from VSW data. With this method, we demonstrated that there is excellent agreement between RHR determined by ECG and VSW, suggesting that the VSW is capable of determining a reliable RHR. In a world where telehealth is increasingly used, reliable wearable device-based data such as this may be useful to clinicians, providing them with clinical information that would otherwise be more cumbersome to obtain [21].

While most studies in the past have focused on analyzing the relationship of RHR with objective, laboratory-based measurements, we also extensively evaluated the relationship of RHR with participants’ well-being and quality of life, including psychosocial and socioeconomic aspects. In the univariate analyses, we demonstrated that participants who had higher education were married, had health care insurance, and had lower PHQ-9 scores were more likely to have a lower VSW RHR. Furthermore, we found similar and significant associations in our regression models when stratified by sex: lack of health care insurance, psychiatric conditions (major depressive disorder and generalized anxiety disorder), and higher WHO-DAS 2.0 scores were significantly associated with higher HR. These findings are consistent with previous studies suggesting that more difficult SES and psychosocial circumstances were associated with higher chronic stress and higher HR [22-28]. However, in our analyses, we also found that there were differences by sex in which baseline characteristics were most associated with RHR. For instance, within the demographics and SES domain, unemployment was most significantly associated with higher RHR for female participants but lack of health care insurance was the most significantly associated with higher RHR for male participants. Similarly, within the PRO domain, a higher WHODAS 2.0 score was most associated with higher RHR for female participants, but the Behavioral Risk Factor Surveillance System Adverse Childhood Experience score was most associated with higher RHR for male participants. This may be the result of a multitude of factors, including physiological differences between the 2 sexes, societal influences, and diverse cultural and personal experiences that could impact HR [29-32]. In the laboratory setting, it has been demonstrated that there are sex differences in HR responses to physical and mental stressors [33-35]. A recent study using a contemporary wearable device investigated the effects of occupational stressors in the real world and found that female participants, compared with male participants, had a higher maximum HR and greater changes in HR when confronted with a moderate stressor during a work shift in a retail store [36]. Future studies will be needed to elucidate the relationships and mechanisms underlying how different clinical characteristics affect RHR in females and males.

We observed significant trends of VSW RHR with objective clinical measurements in both our univariate and regression analyses. Higher VSW RHR was associated with higher blood pressure, BMI, and waist circumference, all previously established in the literature [37,38]. Laboratory findings of higher C-reactive protein and platelet counts in those participants with higher SW RHR were also consistent with the literature [39]. Analyses of physical function showed significant trends with VSW RHR. Lower VSW RHR was significantly correlated with a higher 6-minute walk distance, an important clinical surrogate for fitness [40]. It has been demonstrated previously that HR profiles determined by wrist-worn devices can predict 6-minute walk distances in patients with mitral or aortic valve disease [11]. Another more commonplace measure of physical activity and fitness is step count, a measure that has been associated with mortality [41]. We observed that participants with lower VSW RHR had significantly higher step counts, consistent with previous studies demonstrating a negative relationship between VSW RHR and physical fitness [28,42,43]. Though causality cannot be determined from these analyses, the relationship between VSW RHR and step count is of high interest to clinicians and patients alike, given step count and other surrogates of physical fitness are integral elements of wearable devices that are often promoted as a method of remote monitoring. Interestingly, the relationship demonstrated in our study was of VSW RHR and future step count, suggesting that even a single RHR measurement could be indicative of a person’s future physical activity and, therefore, may identify a population with higher RHR for targeted interventions aimed to improve physical fitness. Future studies will need to longitudinally track both RHR and physical activity levels to determine if their long-term trends are indeed correlated.

There are several limitations to our analysis. Our cohort may have a slight healthy user bias, given it was derived from the PBHS registry, and this potential self-selection bias may have had an impact on some of our findings, such as the differences across sexes. The analysis cohort was also more limited in size than expected, primarily due to a lack of procedural consistency (wearing the VSW at the time of ECG recording) during the participant enrollment visit, resulting in a loss of ~50% of participants from the DPC cohort. It is possible that this can contribute further to a healthy user bias, as those who are more proactive in wearing the VSW may also have been healthier. Future studies will need to ensure more rigid protocols to ensure less variability due to procedural issues. In this study, hard clinical outcomes such as mortality and hospitalizations were not assessed but would be highly valuable for future studies, particularly those that evaluate not only associations of RHR with clinical outcomes but also of “free-living” HR with clinical outcomes. Other studies have examined the validity of using wearable devices to measure HR under free-living conditions, which is currently under investigation in the PBHS [44,45]. Finally, there was a positive bias of 0.76 BPM (95% CI 0.52-1.00) in VSW RHR measurements compared with the reference ECG RHR. This bias changed significantly as a function of ECG RHR, with a negative slope of –0.029 (95% CI –0.047 to –0.010). The most likely cause for the bias is the relative noisiness of PPG signals (measured by SW) compared with ECG, which occasionally results in the detection of false beats. While both bias and slope values are statistically significant, they are unlikely to be clinically meaningful.

### Conclusion

In conclusion, VSW RHR correlates strongly with RHR obtained using resting ECG. VSW RHR has significant trends with important clinical characteristics that closely mirror those already established in the literature. Further investigations will be needed to inform clinicians and patients alike on how to use wearable technologies that perform noninvasive measurements—not only of RHR—in conjunction with other clinical measurements to potentially detect disease or enhance their shared decision-making process for behavioral change.

## Appendix

Multimedia Appendix 1 Methods and results.

Multimedia Appendix 2 High-resolution version of Figure 5.

## Abbreviations

CVD: cardiovascular disease
ECG: electrocardiography
ENET: Elastic net
HR: heart rate
IBI: interbeat interval
IRB: institutional review board
ICC: intraclass correlation coefficient
LASSO: least absolute shrinkage and selection operator
PBHS: Project Baseline Health Study
PHQ-9: patient health questionnaire-9
PPG: photoplethysmography
PRO: patient-reported outcome
RHR: resting heart rate
SES: socioeconomic status
VSW: Verily Study Watch
WHODAS: World Health Organization Disability Assessment Schedule

## Data Availability

The deidentified PBHS data corresponding to this study are available upon request for the purpose of examining its reproducibility. Interested investigators should direct requests to jsaiz@verily.com. Requests are subject to approval by PBHS governance.
