# Supplementary material for: Resting Heart Rate and Associations With Clinical Measures From the Project Baseline Health Study: Observational Study
Source: J Med Internet Res. 2024 Dec 20;26:e60493. doi: 10.2196/60493 (PMC11699500; doi:10.2196/60493)
Supplement: Multimedia Appendix 1 [file jmir_v26i1e60493_app1.docx]

# MULTIMEDIA APPENDIX

# Resting Heart Rate and Associations with Clinical Measures from the Project Baseline Health Study

Kent Y. Feng, MD; Sarah A. Short, MPH; Sohrab Saeb, PhD; Megan K. Carroll, MS; Christoph B. Olivier, MD; Edgar P. Simard, PhD, MPH; Sue Swope; Donna Williams; Julie Eckstrand, RPh; Neha Pagidipati, MD, MPH; Svati H. Shah, MD, MHS; Adrian F. Hernandez, MD; Kenneth W. Mahaffey, MD; on behalf of the Project Baseline Health Study Group

# METHODS AND RESULTS

## Stratified Enrollment by Risk [9]

Baseline Registry participants were selected to obtain a study population enriched for participants with an elevated risk of cardiovascular disease (CVD), lung cancer, and breast/ovarian cancers.

### *Elevated Risk for Cardiovascular Disease*

Within each age strata, 60% of the total enrolled population were to meet all of the following criteria for elevated CVD risk:

- No prior or current atherosclerotic cardiovascular disease (ASCVD); including prior myocardial infarction, stroke, surgery, amputations, significant blockages, or stent placed for blockages in the carotids, coronaries, or peripherals as reported by the participant.
- No current cancer as reported by the participant.
- Upper 60th percentile of risk scores for CVD risk relative to the distribution of risks for the same age and sex observed in the interview sample population of the 2011-2012 NHANES survey. Participants risks and NHANES survey risks are calculated using published risk scores as follows:
  - Framingham Risk Score for 30-year risk of hard CVD events for those ages 18-39 years.
  - 2013 American College of Cardiology/American Heart Association (ACC/AHA) ASCVD risk estimation equation for 10-year risk for hard CVD events for those ages 40+ years.

### *Elevated Risk for Lung Cancer*

Within each age strata, 60% of the total enrolled population were to meet all of the following criteria for elevated risk of lung cancer:

- No prior or current cancer as reported by the participant (except non-melanoma skin cancer).
- Must meet at least one of these criteria for elevated lung cancer risk:
  - Upper 60th percentile of lung cancer risk relative to the distribution of cancer risks for the same age and sex observed in the subpopulation of smokers in the 2010 NHIS Cancer Control Supplement (CCS).
  - Current cigarette smoking with cumulative ≥ 30 pack year history.
  - Previous cumulative cigarette smoking history ≥ 30 pack year and quit < 15 years prior to enrollment.

### *Elevated Risk for Breast/Ovarian Cancers*

Within each age strata, 60% of the total enrolled female population were to meet all of the following criteria for elevated risk of breast/ovarian cancers:

- No prior or current cancer as reported by the participant (except non-melanoma skin cancer).
- No prophylactic cancer surgery (bilateral mastectomy, risk-reducing salpingo- oophorectomy) as reported by the participant.
- Must meet at least one of these criteria for elevated breast/ovarian cancer risk:
  - Upper 60th percentile of breast/ovarian cancers risk relative to the distribution of cancer risk for the same age and sex observed in the 2010 NHIS CCS.
  - Known carrier of a genetic mutation associated with breast/ovarian cancers (BRCA1, BRCA2).

## Participants with an elevated breast/ovarian cancer risk will be female, reflecting conventional risk model’s definition of breast/ovarian cancer risk only among females.

## Table S1. Analysis cohort: Demographics and socioeconomic status at baseline.

### a. female participants

|  | **VSW RHR percentile*** | | | |
| --- | --- | --- | --- | --- |
|  | **0-25th pctle**  **(n = 130)** | **25-75th pctle**  **(n = 259)** | **75-100th pctle**  **(n = 130)** | ***P*-value** |
| Mean age, yrs (SD) | 48.7 (16.3) | 51.1 (16.1) | 49.3 (15.8) | .7635 |
| Race, n (%) |  |  |  |  |
| White | 93 (71.5) | 164 (63.3) | 83 (63.8) | .1924 |
| Black | 18 (13.8) | 44 (17.0) | 26 (20.0) | .1865 |
| Asian | 10 (7.7) | 27 (10.4) | 6 (4.6) | .3686 |
| Native Hawaiian Pacific Islander | 0 (0.0) | 2 (0.8) | 1 (0.8) | .7033 |
| American Indian or Alaska Native | 1 (0.8) | 5 (1.9) | 3 (2.3) | .3425 |
| Other race | 8 (6.2) | 17 (6.6) | 11 (8.5) | .4644 |
| Hispanic ethnicity, n (%) | 14 (10.8) | 40 (15.4) | 17 (13.1) | .5886 |
| Site, n (%) |  |  |  |  |
| Los Angeles | 39 (30.0) | 59 (22.8) | 12 (9.2) | <.001 |
| Durham | 44 (33.8) | 71 (27.4) | 29 (22.3) | .0379 |
| Kannapolis | 30 (23.1) | 62 (23.9) | 57 (43.8) | <.001 |
| Palo Alto | 17 (13.1) | 67 (25.9) | 32 (24.6) | .0257 |
| Education, n (%) |  |  |  |  |
| High school or less | 11 (9.7) | 21 (9.5) | 24 (20.9) | .0107 |
| Some college | 25 (22.1) | 64 (29.0) | 38 (33.0) | .0678 |
| College | 40 (35.4) | 79 (35.7) | 28 (24.3) | .0744 |
| Graduate degree or higher | 37 (32.7) | 57 (25.8) | 25 (21.7) | .0604 |
| Income, n (%) |  |  |  |  |
| < $25,000 | 10 (8.8) | 21 (9.5) | 26 (22.6) | .0018 |
| $25,000-50,000 | 18 (15.9) | 35 (15.8) | 19 (16.5) | .9025 |
| $50,000-100,000 | 30 (26.5) | 67 (30.3) | 37 (32.2) | .3546 |
| $100,000-150,000 | 19 (16.8) | 26 (11.8) | 8 (7.0) | .0212 |
| $150,000-200,000 | 14 (12.4) | 21 (9.5) | 7 (6.1) | .1025 |
| > $200,000 | 13 (11.5) | 35 (15.8) | 10 (8.7) | .5204 |
| Marital status, n (%) |  |  |  |  |
| Married | 63 (55.8) | 131 (59.3) | 50 (43.5) | .0614 |
| Living together | 7 (6.2) | 15 (6.8) | 8 (7.0) | .8185 |
| Divorced | 16 (14.2) | 29 (13.1) | 16 (13.9) | .9582 |
| Separated | 5 (4.4) | 7 (3.2) | 6 (5.2) | .7564 |
| Widowed | 3 (2.7) | 7 (3.2) | 10 (8.7) | .0267 |
| Formerly in long term relationship | 3 (2.7) | 9 (4.1) | 3 (2.6) | .9804 |
| Single | 24 (21.2) | 43 (19.5) | 33 (28.7) | .1738 |
| Never in long term relationship | 14 (12.4) | 19 (8.6) | 22 (19.1) | .1176 |
| Employment status, n (%) |  |  |  |  |
| Employed for wages | 68 (52.3) | 121 (46.7) | 60 (46.2) | .3211 |
| Self-employed | 18 (13.8) | 26 (10.0) | 6 (4.6) | .0117 |
| Homemaker | 6 (4.6) | 17 (6.6) | 3 (2.3) | .3942 |
| Retired | 19 (14.6) | 52 (20.1) | 23 (17.7) | .5199 |
| Student | 5 (3.8) | 4 (1.5) | 2 (1.5) | .1969 |
| Not working >= 1 year | 2 (1.5) | 6 (2.3) | 6 (4.6) | .1261 |
| Not working < 1 year | 3 (2.3) | 6 (2.3) | 7 (5.4) | .1516 |
| Unable to work | 1 (0.8) | 5 (1.9) | 11 (8.5) | <.001 |
| Health insurance, n (%) |  |  |  |  |
| Insured | 109 (96.5) | 202 (91.4) | 101 (89.4) | .0479 |
| Smoking status, n (%) |  |  |  |  |
| Current smoker | 12 (9.2) | 41 (15.8) | 31 (23.8) | .0014 |
| Former smoker | 27 (20.8) | 54 (20.8) | 24 (18.5) | .6436 |
| Never smoker | 91 (70.0) | 164 (63.3) | 75 (57.7) | .0394 |

* Percentile cutpoints for females: 25th = 59.38 bpm; 75th = 73.66 bpm. Shading for significant observations.
RHR=resting heart rate; SD=standard deviation; VSW=Verily study watch

### b. male participants

|  | **VSW RHR percentile*** | | | |
| --- | --- | --- | --- | --- |
|  | **0-25th pctle**  **(n = 89)** | **25-75th pctle**  **(n = 178)** | **75-100th pctle**  **(n = 89)** | ***P*-value** |
| Mean age, yrs (SD) | 55.5 (16.6) | 50.7 (18.5) | 51.6 (14.3) | .1055 |
| Race, n (%) |  |  |  |  |
| White | 71 (79.8%) | 107 (60.1%) | 57 (64.0%) | .027 |
| Black | 11 (12.4%) | 24 (13.5%) | 15 (16.9%) | .3889 |
| Asian | 4 (4.5%) | 24 (13.5%) | 9 (10.1%) | .2201 |
| Native Hawaiian Pacific Islander | 0 (0.0%) | 3 (1.7%) | 1 (1.1%) | .7346 |
| American Indian or Alaska Native | 0 (0.0%) | 2 (1.1%) | 1 (1.1%) | .7019 |
| Other race | 3 (3.4%) | 18 (10.1%) | 6 (6.7%) | .3964 |
| Hispanic ethnicity, n (%) | 6 (6.7%) | 12 (6.7%) | 9 (10.1%) | .3964 |
| Site, n (%) |  |  |  |  |
| Los Angeles | 20 (22.5%) | 37 (20.8%) | 14 (15.7%) | .2611 |
| Durham | 30 (33.7%) | 42 (23.6%) | 18 (20.2%) | .0388 |
| Kannapolis | 24 (27.0%) | 53 (29.8%) | 32 (36.0%) | .1939 |
| Palo Alto | 15 (16.9%) | 46 (25.8%) | 25 (28.1%) | .0804 |
| Education, n (%) |  |  |  |  |
| High school or less | 7 (9.7%) | 16 (10.7%) | 12 (15.4%) | .2757 |
| Some college | 17 (23.6%) | 32 (21.5%) | 30 (38.5%) | .0348 |
| College | 17 (23.6%) | 42 (28.2%) | 18 (23.1%) | .919 |
| Graduate degree or higher | 31 (43.1%) | 59 (39.6%) | 18 (23.1%) | .01 |
| Income, n (%) |  |  |  |  |
| < $25,000 | 6 (8.3%) | 11 (7.4%) | 8 (10.3%) | .6589 |
| $25,000-50,000 | 10 (13.9%) | 29 (19.5%) | 11 (14.1%) | .9994 |
| $50,000-100,000 | 18 (25.0%) | 40 (26.8%) | 22 (28.2%) | .6591 |
| $100,000-150,000 | 16 (22.2%) | 18 (12.1%) | 12 (15.4%) | .2658 |
| $150,000-200,000 | 4 (5.6%) | 13 (8.7%) | 7 (9.0%) | .4498 |
| > $200,000 | 15 (20.8%) | 33 (22.1%) | 9 (11.5%) | .1381 |
| Marital status, n (%) |  |  |  |  |
| Married | 57 (79.2%) | 86 (57.7%) | 46 (59.0%) | .0124 |
| Living together | 2 (2.8%) | 15 (10.1%) | 9 (11.5%) | .0608 |
| Divorced | 4 (5.6%) | 11 (7.4%) | 5 (6.4%) | .8451 |
| Separated | 1 (1.4%) | 0 (0.0%) | 2 (2.6%) | .4804 |
| Widowed | 0 (0.0%) | 1 (0.7%) | 2 (2.6%) | .2768 |
| Formerly in long term relationship | 2 (2.8%) | 4 (2.7%) | 7 (9.0%) | .0581 |
| Single | 9 (12.5%) | 49 (32.9%) | 22 (28.2%) | .0358 |
| Never in long term relationship | 5 (6.9%) | 30 (20.1%) | 6 (7.7%) | .9665 |
| Employment status, n (%) |  |  |  |  |
| Employed for wages | 32 (36.0%) | 83 (46.6%) | 43 (48.3%) | .0975 |
| Self-employed | 11 (12.4%) | 20 (11.2%) | 12 (13.5%) | .8183 |
| Homemaker | 0 (0.0%) | 0 (0.0%) | 2 (2.2%) | .2181 |
| Retired | 28 (31.5%) | 40 (22.5%) | 16 (18.0%) | .0344 |
| Student | 0 (0.0%) | 4 (2.2%) | 1 (1.1%) | .7582 |
| Not working >= 1 year | 2 (2.2%) | 2 (1.1%) | 4 (4.5%) | .3125 |
| Not working < 1 year | 3 (3.4%) | 4 (2.2%) | 1 (1.1%) | .3125 |
| Unable to work | 4 (4.5%) | 3 (1.7%) | 6 (6.7%) | .4248 |
| Health insurance, n (%) |  |  |  |  |
| Insured | 69 (95.8%) | 137 (91.9%) | 67 (85.9%) | .0304 |
| Smoking status, n (%) |  |  |  |  |
| Current smoker | 16 (18.0%) | 22 (12.4%) | 17 (19.1%) | .8359 |
| Former smoker | 21 (23.6%) | 41 (23.0%) | 25 (28.1%) | .486 |
| Never smoker | 52 (58.4%) | 115 (64.6%) | 47 (52.8%) | .4447 |

* Percentile cutpoints for males: 25th = 55.50 bpm; 75th = 72.25 bpm. Shading for significant observations.
RHR=resting heart rate; SD=standard deviation; VSW=Verily study watch

## Table S2. Analysis cohort: Vitals, physical function, and labs at baseline.

### a. female participants

|  | **VSW RHR percentile*** | | | |
| --- | --- | --- | --- | --- |
|  | **0-25th pctle**  **(n = 130)** | **25-75th pctle**  **(n = 259)** | **75-100th pctle**  **(n = 130)** | ***P*-value** |
| Vitals, mean (SD) |  |  |  |  |
| Systolic blood pressure | 119.5 (15.2) | 122.6 (15.5) | 126.5 (15.6) | .0002 |
| Diastolic blood pressure | 73.2 (8.3) | 76.0 (9.2) | 81.1 (10.0) | <.001 |
| Waist circumference | 85.3 (14.4) | 89.8 (15.9) | 98.5 (18.0) | <.001 |
| BMI | 27.1 (6.4) | 28.5 (6.7) | 32.9 (8.4) | <.001 |
| Oxygen saturation | 99.0 (1.2) | 98.8 (1.4) | 98.2 (1.4) | <.001 |
| Respiratory rate | 15.4 (1.9) | 15.7 (2.4) | 16.0 (2.2) | .0236 |
| Physical function, mean (SD) |  |  |  |  |
| 6-minute walk | 498.1 (82.7) | 469.3 (81.9) | 433.8 (93.0) | <.001 |
| 10-meter walk speed | 2.0 (0.6) | 1.9 (0.4) | 1.8 (0.5) | .0086 |
| Handgrip | 28.9 (6.9) | 28.1 (6.9) | 27.4 (7.0) | .2264 |
| Mean leg balance time | 44.3 (20.6) | 39.8 (22.1) | 37.8 (23.1) | .0159 |
| Sit-rise score | 7.5 (2.3) | 6.9 (2.5) | 7.0 (2.4) | .1137 |
| 30 second chair stand | 14.8 (4.7) | 13.9 (5.0) | 12.9 (4.3) | .0016 |
| Left ventricular mass index | 65.3 (15.3) | 66.1 (15.1) | 67.8 (18.4) | .5469 |
| Ejection fraction at rest | 59.0 (3.6) | 59.4 (4.3) | 58.5 (5.4) | .3258 |
| Coronary calcium score | 66.6 (214.3) | 60.9 (250.0) | 76.6 (249.1) | .0289 |
| Ankle brachial index abnormal, n (%) | 4 (3.1) | 10 (3.9) | 3 (2.5) | .7888 |
| FEV1/FVC | 0.8 (0.1) | 0.8 (0.1) | 0.8 (0.1) | .2469 |
| Mean daily steps in first 30 days | 8360.0 (2989.9) | 8039.6 (3186.9) | 6865.4 (3243.0) | <.001 |
| Labs, mean (SD) |  |  |  |  |
| Hemoglobin, g/dL | 13.5 (1.0) | 13.5 (1.2) | 13.7 (1.2) | .099 |
| Serum creatinine, mg/dL | 0.8 (0.1) | 0.8 (0.2) | 0.8 (0.2) | .1922 |
| HDL, mg/dL | 66.4 (18.4) | 64.3 (20.5) | 57.2 (14.4) | <.001 |
| LDL, mg/dL | 96.0 (29.1) | 105.5 (33.9) | 105.8 (30.4) | .0175 |
| Triglycerides, mg/dL | 104.2 (54.5) | 127.1 (74.0) | 150.2 (92.8) | <.001 |
| HbA1c, % | 5.4 (0.7) | 5.6 (0.8) | 6.0 (1.5) | <.001 |
| Lymphocytes, thousand/uL | 1.9 (0.6) | 1.9 (0.6) | 2.0 (0.7) | .0442 |
| Neutrophils, thousand/uL | 3.8 (1.4) | 4.0 (1.5) | 4.8 (1.8) | <.001 |
| ALT, ul | 16.3 (6.7) | 17.9 (10.2) | 21.8 (15.8) | .0041 |
| AST, ul | 19.7 (5.4) | 20.7 (13.4) | 20.3 (10.4) | .1045 |
| Vitamin D, ng/mL | 32.9 (13.5) | 30.3 (13.6) | 27.6 (13.3) | .0007 |
| C-reactive protein, mg/L | 2.3 (4.1) | 3.3 (5.1) | 5.6 (8.2) | <.001 |
| Blood glucose, mg/dL | 88.7 (19.0) | 94.1 (27.2) | 108.9 (54.6) | <.001 |
| Neutrophil segments, % WBC | 60.1 (9.1) | 60.2 (8.6) | 62.9 (9.4) | .0199 |
| Magnesium, mEq/L | 1.7 (0.1) | 1.7 (0.2) | 1.7 (0.2) | .1883 |
| Hematocrit, % | 41.2 (2.9) | 41.2 (3.4) | 42.0 (3.5) | .0465 |
| MCV, fl | 91.9 (6.4) | 91.2 (6.9) | 91.0 (6.0) | .0841 |
| MCH, pg | 30.1 (2.4) | 29.8 (2.6) | 29.7 (2.3) | .0694 |
| MPV, fl | 9.5 (1.1) | 9.4 (1.0) | 9.4 (0.9) | .4676 |
| Platelets, per uL | 249836.1 (56779.2) | 259269.4 (61123.8) | 278789.9 (61888.6) | <.001 |
| RBC count, million/uL | 4.5 (0.3) | 4.5 (0.4) | 4.6 (0.4) | .0102 |
| WBC count, thousand/uL | 6.2 (1.6) | 6.5 (1.8) | 7.5 (2.2) | <.001 |
| Calcium, mg/dL | 9.5 (0.3) | 9.5 (0.4) | 9.5 (0.4) | .778 |
| Cholesterol, mg/dL | 183.2 (33.4) | 195.2 (39.8) | 193.6 (39.1) | .1223 |
| Chloride, mEq/L | 103.4 (2.2) | 103.1 (2.4) | 102.8 (2.4) | .0745 |
| Potassium, mEq/L | 4.3 (0.3) | 4.2 (0.4) | 4.2 (0.3) | .0489 |
| Sodium, mEq/L | 139.0 (1.8) | 138.8 (2.1) | 138.6 (2.2) | .1538 |
| Serum protein, g/dL | 6.9 (0.3) | 7.0 (0.4) | 7.1 (0.4) | .0038 |
| Uric acid, mg/dL | 4.4 (1.0) | 4.6 (1.2) | 5.0 (1.2) | <.001 |
| Monocytes, thousand/uL | 0.4 (0.1) | 0.4 (0.1) | 0.4 (0.2) | .0114 |
| Absolute eosinophils, thousand/uL | 0.1 (0.1) | 0.2 (0.1) | 0.2 (0.2) | .6236 |
| Absolute basophils, thousand/uL | 0.0 (0.0) | 0.0 (0.0) | 0.1 (0.0) | .0507 |
| Urine creatinine, mg/L | 82.3 (69.0) | 92.9 (72.9) | 101.6 (68.6) | .0036 |
| GFR MDRD, ml/min | 86.2 (19.2) | 87.5 (20.1) | 91.0 (24.9) | .1876 |
| Absolute reticulocytes, billion/L | 46.3 (18.5) | 52.6 (20.0) | 62.4 (21.8) | <.001 |
| TSH, mIU/L | 1.5 (0.8) | 1.6 (1.2) | 1.6 (0.9) | .3462 |
| Urine reaction pH | 6.5 (0.8) | 6.4 (0.8) | 6.2 (0.6) | .006 |
| Urine specific gravity | 1.0 (0.0) | 1.0 (0.0) | 1.0 (0.0) | .0021 |
| Albumin in urine, g/dL | 4.4 (0.3) | 4.3 (0.3) | 4.3 (0.3) | .0381 |

* Percentile cutpoints for females: 25th = 59.38 bpm; 75th = 73.66 bpm. Shading for significant observations.
BMI=body mass index; FEV1/FVC=forced expiratory volume in 1 s /forced vital capacity; HbA1c=glycated hemoglobin A1c; HDL=high-density lipoprotein; LDL=low-density lipoprotein; GFR MDRD=glomerular filtration rate, modification of diet in renal disease; RHR=resting heart rate; SD=standard deviation; TSH=thyroid-stimulating hormone; VSW=Verily study watch; WBC=white blood cell.

### b. male participants

|  | **VSW RHR percentile*** | | | |
| --- | --- | --- | --- | --- |
|  | **0-25th pctle**  **(n = 89)** | **25-75th pctle**  **(n = 178)** | **75-100th pctle**  **(n = 89)** | ***P*-value** |
| Vitals, mean (SD) |  |  |  |  |
| Systolic blood pressure | 127.7 (16.2) | 128.1 (14.1) | 129.3 (14.3) | .3677 |
| Diastolic blood pressure | 76.1 (10.4) | 78.3 (9.7) | 81.2 (10.1) | .0015 |
| Waist circumference | 95.5 (12.3) | 98.7 (15.7) | 108.4 (18.5) | <.001 |
| BMI | 27.6 (4.5) | 29.3 (5.7) | 32.5 (8.5) | <.001 |
| Oxygen saturation | 98.1 (1.4) | 98.1 (1.5) | 97.4 (1.6) | .0071 |
| Respiratory rate | 15.8 (2.4) | 16.2 (2.2) | 15.9 (2.4) | .5825 |
| Physical function, mean (SD) |  |  |  |  |
| 6-minute walk | 501.5 (83.1) | 490.2 (89.8) | 465.2 (84.6) | .0017 |
| 10-meter walk speed | 2.1 (0.6) | 2.1 (0.6) | 1.9 (0.5) | .0211 |
| Handgrip | 46.0 (9.4) | 44.5 (10.6) | 42.4 (10.3) | .0879 |
| Mean leg balance time | 38.4 (22.8) | 37.7 (23.1) | 30.6 (22.6) | .0117 |
| Sit-rise score | 7.5 (2.1) | 7.0 (2.3) | 6.7 (2.3) | .0152 |
| 30 second chair stand | 15.4 (5.3) | 14.9 (5.5) | 13.4 (4.4) | .0015 |
| Left ventricular mass index | 79.7 (18.9) | 76.9 (20.1) | 73.7 (16.2) | .0396 |
| Ejection fraction at rest | 58.2 (3.7) | 57.7 (4.7) | 58.7 (4.4) | .7558 |
| Coronary calcium score | 361.8 (1012.1) | 254.6 (653.9) | 209.5 (632.9) | .9963 |
| Ankle brachial index abnormal, n (%) | 7 (3.9%) | 2 (2.3%) | 0.6548 |  |
| FEV1/FVC | 0.7 (0.1) | 0.8 (0.1) | 0.8 (0.1) | .0034 |
| Mean daily steps in first 30 days | 8970.4 (3994.3) | 8564.8 (3537.3) | 7868.9 (4119.9) | .0746 |
| Labs, mean (SD) |  |  |  |  |
| Hemoglobin, g/dL | 14.8 (0.9) | 14.9 (1.0) | 15.1 (1.1) | .0247 |
| Serum creatinine, mg/dL | 1.0 (0.2) | 1.0 (0.3) | 1.1 (0.5) | .9464 |
| HDL, mg/dL | 54.3 (17.4) | 48.3 (15.4) | 43.9 (12.8) | <.001 |
| LDL, mg/dL | 93.3 (36.3) | 95.0 (33.1) | 101.6 (38.5) | .2222 |
| Triglycerides, mg/dL | 120.7 (79.3) | 163.5 (159.5) | 213.5 (155.7) | <.001 |
| HbA1c, % | 5.5 (0.5) | 5.7 (1.0) | 6.5 (1.9) | .0034 |
| Lymphocytes, thousand/uL | 1.8 (0.6) | 1.8 (0.5) | 1.9 (0.6) | .0584 |
| Neutrophils, thousand/uL | 3.7 (1.4) | 3.7 (1.3) | 4.3 (1.5) | .0171 |
| ALT, ul | 21.6 (10.5) | 24.6 (13.3) | 25.9 (18.5) | .1118 |
| AST, ul | 21.3 (6.7) | 22.0 (7.7) | 21.8 (10.9) | .8024 |
| Vitamin D, ng/mL | 32.4 (12.6) | 28.0 (12.5) | 24.5 (10.3) | <.001 |
| C-reactive protein, mg/L | 3.0 (14.4) | 2.5 (4.5) | 4.6 (7.3) | <.001 |
| Blood glucose, mg/dL | 92.2 (12.4) | 102.0 (35.9) | 130.1 (72.8) | <.001 |
| Neutrophil segments, % WBC | 59.2 (8.6) | 59.2 (9.6) | 60.9 (9.9) | .291 |
| Magnesium, mEq/L | 1.7 (0.1) | 1.7 (0.1) | 1.7 (0.2) | .0841 |
| Hematocrit, % | 44.7 (2.9) | 45.2 (3.1) | 45.8 (3.2) | .0126 |
| MCV, fl | 92.3 (4.7) | 91.9 (4.9) | 91.8 (6.0) | .5495 |
| MCH, pg | 30.5 (1.8) | 30.3 (1.9) | 30.2 (2.2) | .3589 |
| MPV, fl | 9.5 (1.0) | 9.3 (1.0) | 9.3 (1.0) | .1886 |
| Platelets, per uL | 212528.7 (48990.5) | 228567.3 (53410.7) | 248264.4 (68745.6) | <.001 |
| RBC count, million/uL | 4.9 (0.4) | 4.9 (0.4) | 5.0 (0.5) | .0058 |
| WBC count, thousand/uL | 6.2 (1.9) | 6.2 (1.5) | 6.9 (1.9) | .0095 |
| Calcium, mg/dL | 9.4 (0.3) | 9.5 (0.4) | 9.6 (0.4) | .0212 |
| Cholesterol, mg/dL | 171.7 (41.3) | 174.0 (39.8) | 182.5 (41.9) | .1222 |
| Chloride, mEq/L | 103.5 (2.0) | 102.9 (2.4) | 102.1 (3.3) | .0014 |
| Potassium, mEq/L | 4.4 (0.4) | 4.3 (0.3) | 4.3 (0.4) | .0281 |
| Sodium, mEq/L | 139.4 (1.7) | 138.9 (2.1) | 138.6 (2.4) | .0218 |
| Serum protein, g/dL | 6.9 (0.4) | 7.0 (0.4) | 7.1 (0.4) | <.001 |
| Uric acid, mg/dL | 5.8 (1.1) | 5.8 (1.2) | 6.1 (1.5) | .2854 |
| Monocytes, thousand/uL | 0.5 (0.2) | 0.4 (0.2) | 0.5 (0.2) | .3219 |
| Absolute eosinophils, thousand/uL | 0.2 (0.2) | 0.2 (0.2) | 0.2 (0.1) | .7135 |
| Absolute basophils, thousand/uL | 0.0 (0.0) | 0.0 (0.0) | 0.1 (0.0) | .0556 |
| Urine creatinine, mg/L | 110.0 (66.9) | 118.4 (71.8) | 129.4 (76.7) | .1052 |
| GFR MDRD, ml/min | 84.3 (16.5) | 88.2 (20.7) | 86.6 (25.2) | .3824 |
| Absolute reticulocytes, billion/L | 50.4 (21.9) | 61.6 (26.0) | 69.5 (24.0) | <.001 |
| TSH, mIU/L | 1.8 (1.0) | 1.9 (1.1) | 1.7 (0.9) | .7056 |
| Urine reaction pH | 6.2 (0.7) | 6.2 (0.7) | 6.1 (0.8) | .0666 |
| Urine specific gravity | 1.0 (0.0) | 1.0 (0.0) | 1.0 (0.0) | <.001 |
| Albumin in urine, g/dL | 4.4 (0.3) | 4.5 (0.3) | 4.4 (0.3) | .0826 |

* Percentile cutpoints for males: 25th = 55.50 bpm; 75th = 72.25 bpm. Shading for significant observations.
BMI=body mass index; FEV1/FVC=forced expiratory volume in 1 s /forced vital capacity; HbA1c=glycated hemoglobin A1c; HDL=high-density lipoprotein; LDL=low-density lipoprotein; GFR MDRD=glomerular filtration rate, modification of diet in renal disease; RHR=resting heart rate; SD=standard deviation; TSH=thyroid-stimulating hormone; VSW=Verily study watch; WBC=white blood cell.

## Table S3. Analysis cohort: Medical conditions and participant-reported outcomes (PROs) at baseline.

### a. female participants

|  | **VSW RHR percentile*** | | | |
| --- | --- | --- | --- | --- |
|  | **0-25th pctle**  **(n= 130)** | **25-75th pctle**  **(n = 259)** | **75-100th pctle**  **(n = 130)** | ***P*-value** |
| Medical history, n (%) |  |  |  |  |
| ADHD | 6 (4.6) | 8 (3.1) | 6 (4.6) | 1 |
| Atrial fibrillation | 1 (0.8) | 1 (0.4) | 0 (0.0) | .6532 |
| Alcohol abuse | 1 (0.8) | 3 (1.2) | 4 (3.1) | .1314 |
| Generalized anxiety disorder | 19 (14.6) | 43 (16.6) | 24 (18.5) | .4047 |
| Arrhythmia | 7 (5.4) | 11 (4.2) | 7 (5.4) | 1 |
| Asthma | 16 (12.3) | 35 (13.5) | 23 (17.7) | .2148 |
| Bipolar disorder | 1 (0.8) | 5 (1.9) | 5 (3.8) | .0853 |
| Benign prostatic hyperplasia | 0 (0.0) | 0 (0.0) | 0 (0.0) | nan |
| Breast cancer, | 2 (1.5) | 8 (3.1) | 6 (4.6) | .1516 |
| Coronary artery disease | 4 (3.1) | 5 (1.9) | 7 (5.4) | .2822 |
| Cataracts | 12 (9.2) | 38 (14.7) | 22 (16.9) | .0731 |
| Colon polyps | 7 (5.4) | 26 (10.0) | 10 (7.7) | .5001 |
| Concussions | 10 (7.7) | 13 (5.0) | 7 (5.4) | .4258 |
| COPD with emphysema | 0 (0.0) | 4 (1.5) | 10 (7.7) | <.001 |
| Major depressive disorder | 15 (11.5) | 43 (16.6) | 29 (22.3) | .0202 |
| Diverticulitis | 1 (0.8) | 5 (1.9) | 2 (1.5) | .615 |
| Diverticulosis | 3 (2.3) | 4 (1.5) | 1 (0.8) | .3145 |
| Diabetes type 1 | 2 (1.5) | 2 (0.8) | 1 (0.8) | .5259 |
| Diabetes type 2 | 6 (4.6) | 27 (10.4) | 26 (20.0) | <.001 |
| Drug abuse | 3 (2.3) | 1 (0.4) | 7 (5.4) | .0853 |
| Epilepsy | 1 (0.8) | 4 (1.5) | 3 (2.3) | .3145 |
| Fibromyalgia | 6 (4.6) | 4 (1.5) | 2 (1.5) | .0991 |
| Gallbladder disease | 6 (4.6) | 15 (5.8) | 14 (10.8) | .0481 |
| GERD | 20 (15.4) | 42 (16.2) | 36 (27.7) | .0113 |
| Glaucoma | 4 (3.1) | 10 (3.9) | 1 (0.8) | .2672 |
| Gout | 0 (0.0) | 0 (0.0) | 3 (2.3) | .0761 |
| Hashimoto’s Disease | 4 (3.1) | 3 (1.2) | 4 (3.1) | 1 |
| Chronic headaches | 0 (0.0) | 5 (1.9) | 6 (4.6) | .0204 |
| Hearing loss | 4 (3.1) | 14 (5.4) | 5 (3.8) | .7634 |
| Hemorrhoids | 2 (1.5) | 16 (6.2) | 4 (3.1) | .5385 |
| Hepatitis B | 1 (0.8) | 0 (0.0) | 1 (0.8) | 1 |
| Hepatitis C | 0 (0.0) | 1 (0.4) | 0 (0.0) | 1 |
| Hypertension | 27 (20.8) | 70 (27.0) | 40 (30.8) | .0677 |
| Myocardial infarction | 1 (0.8) | 2 (0.8) | 3 (2.3) | .2464 |
| Hypercholesterolemia | 14 (10.8) | 40 (15.4) | 13 (10.0) | .8534 |
| Hypothyroidism | 14 (10.8) | 27 (10.4) | 18 (13.8) | .4349 |
| Irritable bowel disorder | 10 (7.7) | 12 (4.6) | 12 (9.2) | .6165 |
| Kidney or bladder stones | 3 (2.3) | 12 (4.6) | 7 (5.4) | .2187 |
| Macular degeneration | 0 (0.0) | 2 (0.8) | 1 (0.8) | .7033 |
| Melanoma skin cancer | 1 (0.8) | 7 (2.7) | 2 (1.5) | .6522 |
| Migraines | 24 (18.5) | 36 (13.9) | 36 (27.7) | .0555 |
| Nonalcoholic fatty liver disease | 1 (0.8) | 1 (0.4) | 4 (3.1) | .0821 |
| Nonmelanoma skin cancer | 12 (9.2) | 24 (9.3) | 7 (5.4) | .2611 |
| Osteoarthritis | 25 (19.2) | 49 (18.9) | 32 (24.6) | .282 |
| Osteopenia | 3 (2.3) | 26 (10.0) | 6 (4.6) | .4586 |
| Osteoporosis | 7 (5.4) | 11 (4.2) | 6 (4.6) | .768 |
| Pulmonary embolism | 2 (1.5) | 2 (0.8) | 3 (2.3) | .5912 |
| Pneumonia | 11 (8.5) | 14 (5.4) | 16 (12.3) | .2508 |
| Prostate cancer | 0 (0.0) | 0 (0.0) | 0 (0.0) | nan |
| Psoriasis | 2 (1.5) | 5 (1.9) | 4 (3.1) | .3896 |
| PTSD | 4 (3.1) | 8 (3.1) | 7 (5.4) | .3223 |
| Peptic ulcer | 4 (3.1) | 2 (0.8) | 6 (4.6) | .4096 |
| Peripheral vascular disease | 1 (0.8) | 3 (1.2) | 3 (2.3) | .2827 |
| Rheumatoid arthritis | 3 (2.3) | 4 (1.5) | 5 (3.8) | .4096 |
| Sleep apnea | 6 (4.6) | 16 (6.2) | 11 (8.5) | .2042 |
| Stroke | 1 (0.8) | 5 (1.9) | 1 (0.8) | 1 |
| Goiter | 3 (2.3) | 5 (1.9) | 1 (0.8) | .3425 |
| Transient ischemic attack | 3 (2.3) | 0 (0.0) | 3 (2.3) | 1 |
| Tinnitus | 3 (2.3) | 13 (5.0) | 5 (3.8) | .5294 |
| PROs, mean score (SD) |  |  |  |  |
| Sheehan Disability Scale | 2.9 (4.5) | 2.7 (4.8) | 5.0 (7.6) | .0971 |
| PHQ-9 | 3.4 (3.6) | 3.6 (4.0) | 5.4 (4.8) | .002 |
| GAD-7 | 3.2 (3.9) | 3.4 (4.1) | 4.1 (4.9) | .2798 |
| WHODAS 2.0 | 2.2 (3.3) | 3.0 (4.4) | 5.0 (6.7) | <.001 |
| BRFSS ACE | 2.2 (2.2) | 2.4 (2.3) | 2.7 (2.6) | .2394 |
| PROMIS pain intensity | 6.0 (2.3) | 6.1 (2.3) | 7.0 (2.8) | .0152 |
| PROMIS pain interference | 10.2 (5.1) | 10.5 (5.2) | 12.2 (6.3) | .0295 |
| PANAS positive affect | 34.7 (6.4) | 34.8 (7.0) | 33.0 (7.3) | .0792 |
| PANAS negative affect | 15.6 (6.7) | 15.5 (6.5) | 15.0 (6.1) | .4481 |
| Subjective Happiness | 21.6 (4.8) | 21.7 (4.8) | 20.9 (4.3) | .1264 |
| Satisfaction with Life | 26.1 (6.6) | 25.9 (6.3) | 24.2 (7.4) | .0437 |
| Perceived Social Support | 70.1 (11.8) | 66.8 (15.2) | 66.9 (13.9) | .0811 |
| AUDIT-C | 2.0 (1.5) | 2.0 (1.9) | 1.9 (1.8) | .3505 |

* Percentile cutpoints for females: 25th = 59.38 bpm; 75th = 73.66 bpm. Shading for significant observations.
AUDIT-C=Alcohol Use Disorders Identification Test-Concise; BRFSS ACE=Behavioral Risk Factor Surveillance System Adverse Childhood Experience ; GAD-7 = general anxiety disorder -7; GERD = gastro-esophageal reflux disease; PHQ-9 = patient health questionnaire -9; PANAS=Positive and Negative Affect Schedule ; PROMIS=Patient-Reported Outcomes Measurement Information System; RHR=resting heart rate; SD = standard deviation; VSW= Verily study watch; WHODAS=World Health Organization Disability Assessment Schedule

### b. male participants

|  | **VSW RHR percentile*** | | | |
| --- | --- | --- | --- | --- |
|  | **0-25th pctle**  **(n = 89)** | **25-75th pctle**  **(n = 178)** | **75-100th pctle**  **(n = 89)** | ***P*-value** |
| Medical history, n (%) |  |  |  |  |
| ADHD | 7 (7.9%) | 18 (10.1%) | 9 (10.1%) | .6105 |
| Atrial fibrillation | 1 (1.1%) | 1 (0.6%) | 0 (0.0%) | .652 |
| Alcohol abuse | 2 (2.2%) | 4 (2.2%) | 2 (2.2%) | 1 |
| Generalized anxiety disorder | 5 (5.6%) | 18 (10.1%) | 12 (13.5%) | .0785 |
| Arrhythmia | 10 (11.2%) | 13 (7.3%) | 0 (0.0%) | .0041 |
| Asthma | 12 (13.5%) | 22 (12.4%) | 16 (18.0%) | .3889 |
| Bipolar disorder | 0 (0.0%) | 4 (2.2%) | 6 (6.7%) | .0148 |
| Benign prostatic hyperplasia | 7 (7.9%) | 14 (7.9%) | 5 (5.6%) | .5651 |
| Breast cancer, | 0 (0.0%) | 0 (0.0%) | 0 (0.0%) | nan |
| Coronary artery disease | 10 (11.2%) | 14 (7.9%) | 5 (5.6%) | .1713 |
| Cataracts | 14 (15.7%) | 28 (15.7%) | 5 (5.6%) | .0466 |
| Colon polyps | 14 (15.7%) | 23 (12.9%) | 6 (6.7%) | .0661 |
| Concussions | 5 (5.6%) | 15 (8.4%) | 7 (7.9%) | .5718 |
| COPD with emphysema | 5 (5.6%) | 7 (3.9%) | 4 (4.5%) | .7179 |
| Major depressive disorder | 8 (9.0%) | 28 (15.7%) | 19 (21.3%) | .0227 |
| Diverticulitis | 2 (2.2%) | 5 (2.8%) | 3 (3.4%) | .6506 |
| Diverticulosis | 2 (2.2%) | 4 (2.2%) | 4 (4.5%) | .3649 |
| Diabetes type 1 | 0 (0.0%) | 1 (0.6%) | 3 (3.4%) | .0933 |
| Diabetes type 2 | 3 (3.4%) | 23 (12.9%) | 27 (30.3%) | <.001 |
| Drug abuse | 1 (1.1%) | 4 (2.2%) | 3 (3.4%) | .3125 |
| Epilepsy | 2 (2.2%) | 3 (1.7%) | 1 (1.1%) | .5609 |
| Fibromyalgia | 0 (0.0%) | 2 (1.1%) | 0 (0.0%) | 1 |
| Gallbladder disease | 5 (5.6%) | 9 (5.1%) | 5 (5.6%) | 1 |
| GERD | 21 (23.6%) | 37 (20.8%) | 20 (22.5%) | .8564 |
| Glaucoma | 0 (0.0%) | 6 (3.4%) | 1 (1.1%) | .7903 |
| Gout | 5 (5.6%) | 11 (6.2%) | 2 (2.2%) | .3054 |
| Hashimoto’s Disease | 1 (1.1%) | 1 (0.6%) | 0 (0.0%) | .652 |
| Chronic headaches | 2 (2.2%) | 1 (0.6%) | 2 (2.2%) | 1 |
| Hearing loss | 9 (10.1%) | 16 (9.0%) | 3 (3.4%) | .0953 |
| Hemorrhoids | 2 (2.2%) | 7 (3.9%) | 1 (1.1%) | .6506 |
| Hepatitis B | 0 (0.0%) | 3 (1.7%) | 1 (1.1%) | .7346 |
| Hepatitis C | 1 (1.1%) | 3 (1.7%) | 1 (1.1%) | 1 |
| Hypertension | 29 (32.6%) | 61 (34.3%) | 35 (39.3%) | .3468 |
| Myocardial infarction | 6 (6.7%) | 9 (5.1%) | 3 (3.4%) | .3054 |
| Hypercholesterolemia | 15 (16.9%) | 27 (15.2%) | 9 (10.1%) | .1999 |
| Hypothyroidism | 5 (5.6%) | 12 (6.7%) | 5 (5.6%) | 1 |
| Irritable bowel disorder | 2 (2.2%) | 5 (2.8%) | 2 (2.2%) | 1 |
| Kidney or bladder stones | 6 (6.7%) | 19 (10.7%) | 13 (14.6%) | .0897 |
| Macular degeneration | 1 (1.1%) | 3 (1.7%) | 0 (0.0%) | .7346 |
| Melanoma skin cancer | 4 (4.5%) | 5 (2.8%) | 0 (0.0%) | .103 |
| Migraines | 4 (4.5%) | 13 (7.3%) | 3 (3.4%) | .7451 |
| Nonalcoholic fatty liver disease | 1 (1.1%) | 3 (1.7%) | 4 (4.5%) | .1298 |
| Nonmelanoma skin cancer | 7 (7.9%) | 14 (7.9%) | 3 (3.4%) | .2325 |
| Osteoarthritis | 21 (23.6%) | 33 (18.5%) | 19 (21.3%) | .7108 |
| Osteopenia | 0 (0.0%) | 0 (0.0%) | 0 (0.0%) | nan |
| Osteoporosis | 1 (1.1%) | 1 (0.6%) | 0 (0.0%) | .652 |
| Pulmonary embolism | 0 (0.0%) | 3 (1.7%) | 3 (3.4%) | .161 |
| Pneumonia | 6 (6.7%) | 10 (5.6%) | 7 (7.9%) | .7608 |
| Prostate cancer | 4 (4.5%) | 6 (3.4%) | 1 (1.1%) | .1944 |
| Psoriasis | 2 (2.2%) | 4 (2.2%) | 3 (3.4%) | .6335 |
| PTSD | 3 (3.4%) | 3 (1.7%) | 3 (3.4%) | 1 |
| Peptic ulcer | 4 (4.5%) | 2 (1.1%) | 4 (4.5%) | 1 |
| Peripheral vascular disease | 1 (1.1%) | 1 (0.6%) | 1 (1.1%) | 1 |
| Rheumatoid arthritis | 0 (0.0%) | 2 (1.1%) | 1 (1.1%) | .7019 |
| Sleep apnea | 9 (10.1%) | 29 (16.3%) | 17 (19.1%) | .0976 |
| Stroke | 3 (3.4%) | 5 (2.8%) | 2 (2.2%) | .6506 |
| Goiter | 0 (0.0%) | 3 (1.7%) | 0 (0.0%) | 1 |
| Transient ischemic attack | 1 (1.1%) | 1 (0.6%) | 1 (1.1%) | 1 |
| Tinnitus | 7 (7.9%) | 8 (4.5%) | 3 (3.4%) | .1718 |
| PROs, mean score (SD) |  |  |  |  |
| Sheehan Disability Scale | 3.3 (6.6) | 3.1 (5.6) | 4.2 (5.7) | .0587 |
| PHQ-9 | 3.2 (4.1) | 3.8 (4.4) | 4.6 (4.4) | .0149 |
| GAD-7 | 2.1 (3.2) | 2.9 (4.0) | 3.9 (4.8) | .0207 |
| WHODAS 2.0 | 2.2 (4.5) | 3.2 (5.2) | 4.4 (5.4) | <.001 |
| BRFSS ACE | 1.6 (1.9) | 1.9 (2.2) | 2.7 (2.6) | .0115 |
| PROMIS pain intensity | 6.1 (2.7) | 6.4 (2.3) | 6.5 (2.5) | .1839 |
| PROMIS pain interference | 10.0 (5.7) | 10.6 (4.7) | 12.4 (6.1) | .0025 |
| PANAS positive affect | 35.5 (7.7) | 33.7 (7.6) | 32.1 (8.1) | .0095 |
| PANAS negative affect | 14.9 (5.7) | 15.2 (5.9) | 15.1 (5.8) | .9349 |
| Subjective Happiness | 22.0 (4.3) | 20.7 (4.8) | 18.9 (4.6) | <.001 |
| Satisfaction with Life | 26.1 (6.6) | 24.7 (6.7) | 22.5 (6.2) | <.001 |
| Perceived Social Support | 65.8 (13.8) | 61.6 (16.7) | 59.3 (14.5) | .0025 |
| AUDIT-C | 2.3 (1.7) | 2.1 (1.8) | 1.8 (1.8) | .0222 |

* Percentile cutpoints for males: 25th = 55.50 bpm; 75th = 72.25 bpm. Shading for significant observations.
AUDIT-C=Alcohol Use Disorders Identification Test-Concise; BRFSS ACE=Behavioral Risk Factor Surveillance System Adverse Childhood Experience ; GAD-7 = general anxiety disorder -7; GERD = gastro-esophageal reflux disease; PHQ-9 = patient health questionnaire -9; PANAS=Positive and Negative Affect Schedule ; PROMIS=Patient-Reported Outcomes Measurement Information System; RHR=resting heart rate; SD = standard deviation; VSW= Verily study watch; WHODAS=World Health Organization Disability Assessment Schedule

### Table S4. Analysis cohort: Selected baseline characteristics of the analysis cohort and the PBHS, stratified by RHR measured by resting electrocardiogram a. female participants.

|  | **ECG RHR percentile** | | |  |
| --- | --- | --- | --- | --- |
|  | **0-25th pctle**  **(n = 130)** | **25-75th pctle**  **(n = 259)** | **75-100th pctle**  **(n = 130)** | ***P*-value** |
| Age (yrs), mean (SD) | 49.6 (16.2) | 50.4 (16.0) | 49.8 (16.2) | .9383 |
| White, n (%) | 93 (71.5) | 162 (62.5) | 85 (65.4) | .2971 |
| Black, n (%) | 15 (11.5) | 49 (18.9) | 24 (18.5) | .1373 |
| Asian, n (%) | 14 (10.8) | 22 (8.5) | 7 (5.4) | .1156 |
| Other race (NHPI, AIAN, Other), n (%) | 8 (6.2) | 26 (10.0) | 14 (10.8) | .1994 |
| Hispanic ethnicity, n (%) | 14 (10.8) | 40 (15.4) | 17 (13.1) | .5886 |
| Married, n (%) | 69 (60.5) | 119 (53.8) | 56 (49.1) | .0843 |
| Employed or homemaker, n (%) | 90 (72.6) | 166 (69.5) | 69 (57.0) | .0099 |
| Current or former smoker, n (%) | 37 (28.5) | 96 (37.1) | 56 (43.1) | .0144 |
| Systolic blood pressure, mean (SD) | 120.2 (15.6) | 122.3 (15.4) | 126.4 (15.4) | <.001 |
| Diastolic blood pressure, mean (SD) | 73.6 (9.0) | 75.9 (9.2) | 80.8 (9.7) | <.001 |
| BMI, mean (SD) | 26.7 (6.0) | 29.1 (7.1) | 32.1 (8.3) | <.001 |
| 6-minute walk, mean (SD) | 500.9 (78.9) | 467.1 (83.8) | 435.1 (92.4) | <.001 |
| Ejection fraction at rest, mean (SD) | 59.3 (3.7) | 59.0 (4.6) | 59.0 (5.0) | .3434 |
| Hemoglobin (g/dL) | 13.4 (1.0) | 13.5 (1.1) | 13.7 (1.4) | .0826 |
| HbA1c (%) | 5.4 (0.5) | 5.7 (0.9) | 6.0 (1.4) | <.001 |
| C-reactive protein (mg/L) | 2.2 (4.0) | 3.3 (4.0) | 5.7 (9.2) | <.001 |
| WBC count (thousand/uL) | 6.2 (1.6) | 6.6 (1.8) | 7.4 (2.2) | <.001 |
| GFR MDRD (ml/min) | 85.0 (18.1) | 88.0 (20.6) | 91.2 (24.6) | .0467 |
| Asthma, n (%) | 15 (11.5) | 34 (13.1) | 25 (19.2) | .0764 |
| Major depressive disorder, n (%) | 16 (12.3) | 39 (15.1) | 32 (24.6) | .008 |
| Diabetes type 2, n (%) | 5 (3.8) | 29 (11.2) | 25 (19.2) | <.001 |
| Generalized anxiety disorder, n (%) | 19 (14.6) | 41 (15.8) | 26 (20.0) | .2434 |
| GERD, n (%) | 20 (15.4) | 43 (16.6) | 35 (26.9) | .0176 |
| Hypertension, n (%) | 26 (20.0) | 69 (26.6) | 42 (32.3) | .0245 |
| Hypercholesterolemia, n (%) | 14 (10.8) | 37 (14.3) | 16 (12.3) | .7117 |
| Migraines, n (%) | 25 (19.2) | 39 (15.1) | 32 (24.6) | .264 |
| Osteoarthritis, n (%) | 23 (17.7) | 53 (20.5) | 30 (23.1) | .282 |
| Sleep apnea, n (%) | 6 (4.6) | 16 (6.2%) | 11 (8.5) | .2042 |
| PHQ-9 score, mean (SD) | 3.2 (3.4) | 3.8 (4.2) | 5.3 (4.7) | <.001 |
| GAD-7 score, mean (SD) | 3.1 (3.7) | 3.5 (4.3) | 4.0 (4.7) | .2547 |

* Percentile cutpoints for females: 25th = 58.68 bpm; 75th = 72.16 bpm. Shading for significant observations.
To generate p-values for tests for trend, the Cochran-Armitage was used to evaluate binary variables, including ‘dummy’ indicator variables created for each level of categorical variables, and Spearman Rank Correlation was used to evaluate continuous variables.
AIAN=American Indians and Alaska Natives ; BMI = body mass index; GFR = glomerular filtration rate; GAD-7 = general anxiety disorder -7; GERD = gastro-esophageal reflux disease; MDRD = modification of diet in renal disease ; PHQ-9 = patient health questionnaire -9; NHPI=Native Hawaiian, and Pacific Islander ; RHR=resting heart rate; SD=standard deviation; VSW=Verily study watch

**b. male participants**

|  | **ECG RHR percentile** | | |  |
| --- | --- | --- | --- | --- |
|  | **0-25th pctle**  **(n = 89)** | **25-75th pctle**  **(n = 178)** | **75-100th pctle**  **(n = 89)** | ***P*-value** |
| Age (yrs), mean (SD) | 55.9 (16.6) | 49.8 (18.3) | 53.0 (14.6) | .2406 |
| White, n (%) | 71 (79.8) | 105 (59.0) | 59 (66.3) | .0579 |
| Black, n (%) | 8 (9.0) | 26 (14.6) | 16 (18.0) | .0848 |
| Asian, n (%) | 5 (5.6) | 25 (14.0) | 7 (7.9) | .6238 |
| Other race (NHPI, AIAN, Other), n (%) | 5 (5.6) | 22 (12.4) | 7 (7.9) | .6105 |
| Hispanic ethnicity, n (%) | 5 (5.6) | 14 (7.9) | 8 (9.0) | .3964 |
| Married, n (%) | 53 (73.6) | 92 (61.3) | 44 (57.1) | .0391 |
| Employed or homemaker, n (%) | 43 (53.1) | 105 (66.5) | 55 (66.3) | .0828 |
| Current or former smoker, n (%) | 36 (40.4%) | 63 (35.4%) | 43 (48.3%) | .2846 |
| Systolic blood pressure, mean (SD) | 126.8 (16.4) | 128.2 (14.1) | 130.0 (14.0) | .0861 |
| Diastolic blood pressure, mean (SD) | 75.6 (10.1) | 78.4 (9.9) | 81.5 (9.8) | <.001 |
| BMI, mean (SD) | 27.3 (4.5) | 29.2 (5.7) | 33.0 (8.3) | <.0011 |
| 6-minute walk, mean (SD) | 502.2 (82.3) | 492.1 (90.3) | 460.6 (82.7) | <.001 |
| Ejection fraction at rest, mean (SD) | 58.0 (3.6) | 57.7 (4.6) | 58.9 (4.6) | .2002 |
| Hemoglobin (g/dL) | 14.7 (0.9) | 14.9 (1.0) | 15.1 (1.2) | .0182 |
| HbA1c (%) | 5.5 (0.5) | 5.8 (1.2) | 6.5 (1.7) | <.001 |
| C-reactive protein (mg/L) | 1.4 (1.3) | 3.5 (11.4) | 4.2 (5.9) | <.001 |
| WBC count (thousand/uL) | 6.1 (1.9) | 6.1 (1.5) | 7.1 (1.9) | <.001 |
| GFR MDRD (ml/min) | 84.1 (16.4) | 88.6 (20.2) | 85.8 (25.8) | .5576 |
| Asthma, n (%) | 15 (16.9) | 21 (11.8) | 14 (15.7) | .8294 |
| Major depressive disorder, n (%) | 7 (7.9) | 30 (16.9) | 18 (20.2) | .0227 |
| Diabetes type 2, n (%) | 4 (4.5) | 22 (12.4) | 27 (30.3) | <.001 |
| Generalized anxiety disorder, n (%) | 7 (7.9) | 16 (9.0) | 12 (13.5) | .2088 |
| GERD, n (%) | 22 (24.7) | 35 (19.7) | 21 (23.6) | .8564 |
| Hypertension, n (%) | 27 (30.3) | 61 (34.3) | 37 (41.6) | .1169 |
| Hypercholesterolemia, n (%) | 17 (19.1) | 25 (14.0) | 9 (10.1) | .0874 |
| Migraines, n (%) | 3 (3.4) | 13 (7.3) | 4 (4.5) | .7451 |
| Osteoarthritis, n (%) | 22 (24.7) | 33 (18.5) | 18 (20.2) | .4584 |
| Sleep apnea, n (%) | 11 (12.4) | 27 (15.2) | 17 (19.1) | .214 |
| PHQ-9 score, mean (SD) | 3.4 (4.4) | 3.7 (4.2) | 4.6 (4.5) | .0304 |
| GAD-7 score, mean (SD) | 2.2 (3.3) | 2.9 (3.8) | 3.8 (5.1) | .1244 |

* Percentile cutpoints for males: 25th = 54.73 bpm; 75th = 70.86 bpm. Shading for significant observations.
To generate p-values for tests for trend, the Cochran-Armitage was used to evaluate binary variables, including ‘dummy’ indicator variables created for each level of categorical variables, and Spearman Rank Correlation was used to evaluate continuous variables.
AIAN=American Indians and Alaska Natives ; BMI = body mass index; GFR = glomerular filtration rate; GAD-7 = general anxiety disorder -7; GERD = gastro-esophageal reflux disease; MDRD = modification of diet in renal disease ; PHQ-9 = patient health questionnaire -9; NHPI=Native Hawaiian, and Pacific Islander ; RHR=resting heart rate; SD=standard deviation; VSW=Verily study watch

## Figure S1: Agreement between SW RHR (blue lines) and ECG RHR (red dots). Participants on the x-axis are sorted by their SW RHR values. The blue line shows the spread (standard deviation) of the distribution of SW heart rate values for each participant that was used to calculate the SW RHR.


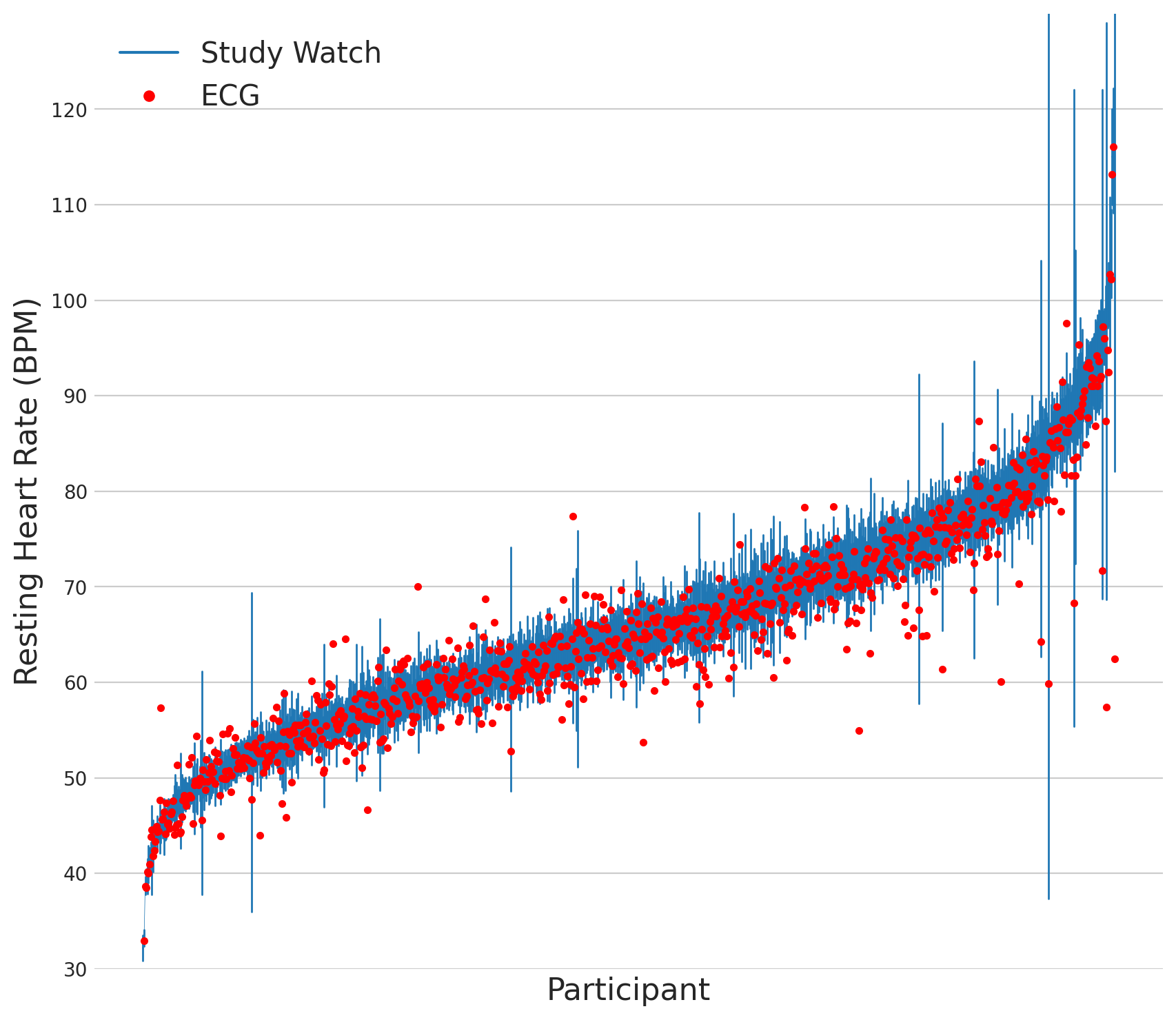


## Figure S2: Difference between SW RHR and ECG RHR as a function of ECG RHR, for (A) all participants, and (B) male and female participants separately. Each blue dot is a single measurement corresponding to a single participant. We have an overall bias of 0.76 BPM (95% CI: 0.52, 1.00) which indicates a small but significant positive bias, meaning that SW is slightly overestimating the RHR when compared to ECG-based RHR. The green line shows a fitted linear model with a slope of -0.029 (95% CI: -0.047, -0.010) which indicates a small but significant negative slope, which implies that for the ECG RHR values on the lower end, SW is overestimating the RHR, while on the higher end it underestimates the RHR relative to the ECG RHR. We have similar results for each male and female subgroups, with a bias of 0.70 (95% CI: 0.29, 1.14) and 0.80 (95% CI: 0.51, 1.13), and a slope of -0.030 (95% CI: -0.057, -0.003) and -0.028 (95% CI: -0.056, -0.003), respectively.

(A)


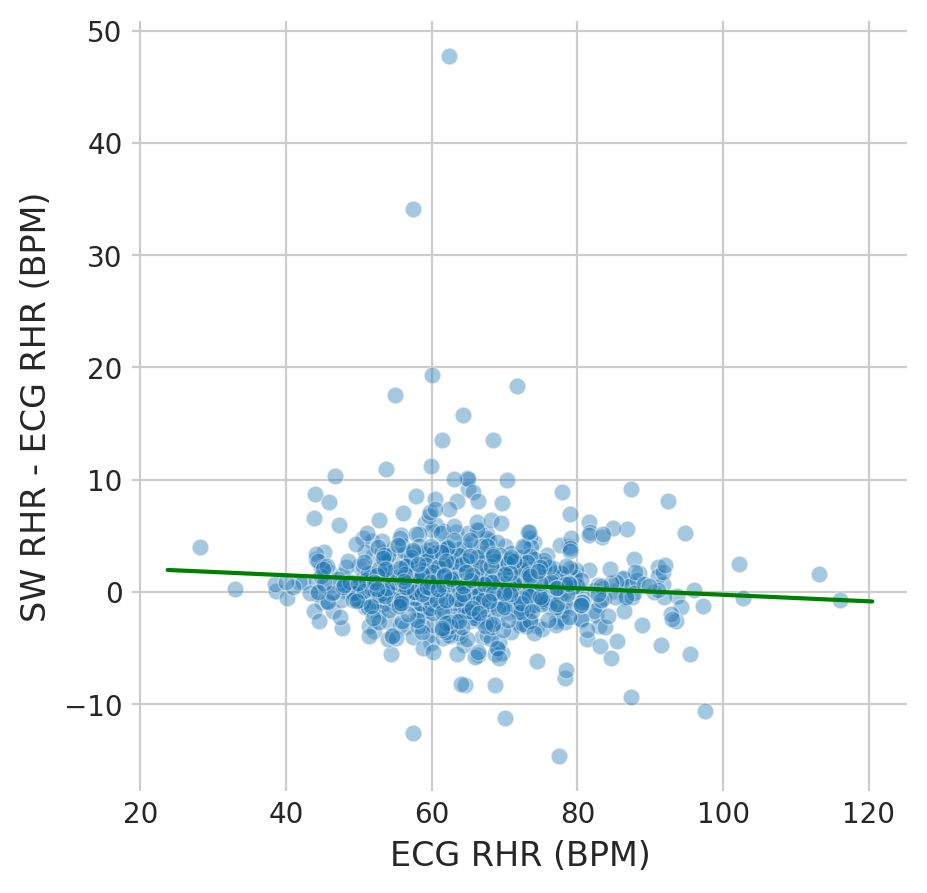


**(B)**

**
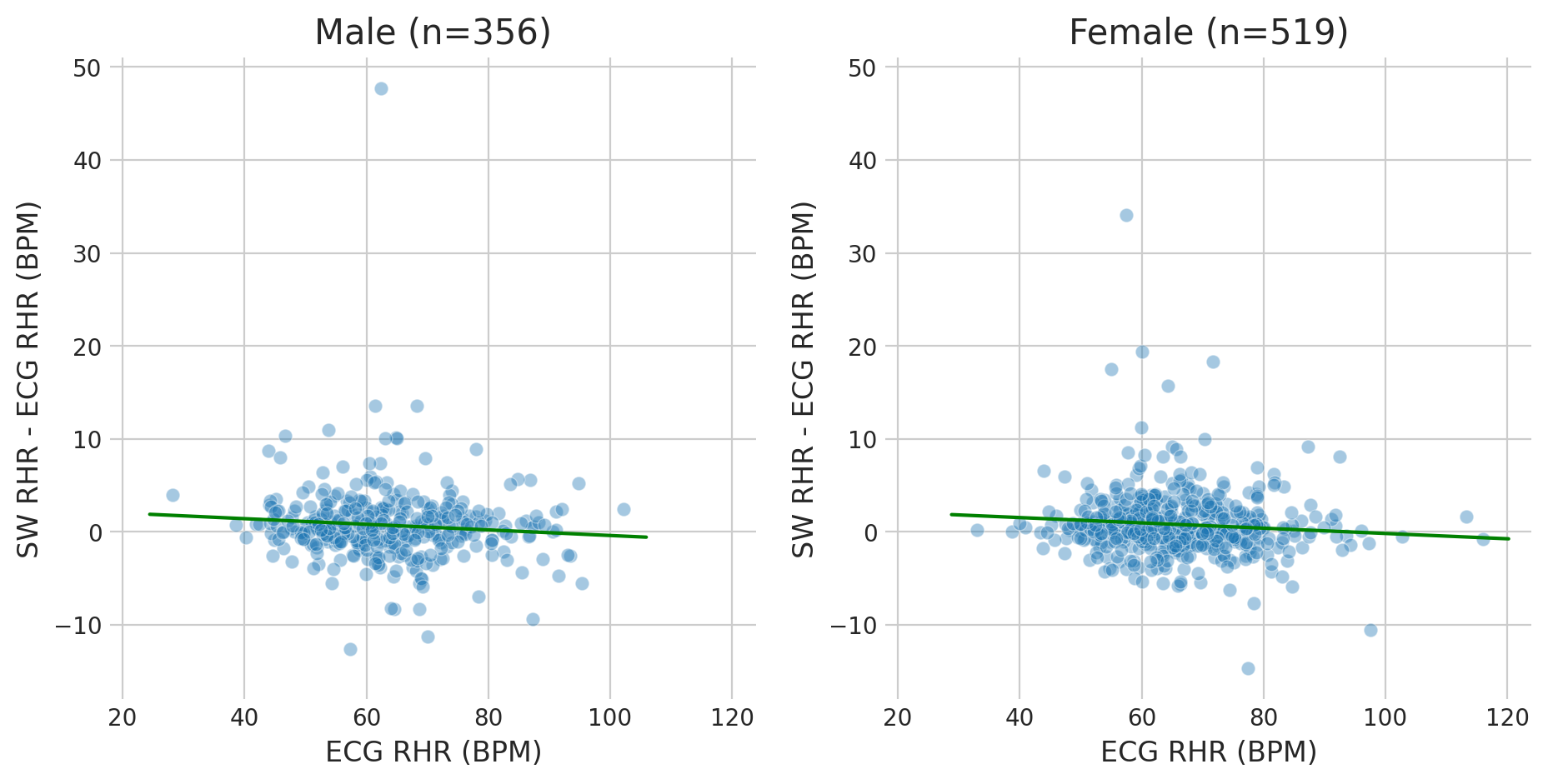
**
